# An open-data-driven agent-based model to simulate infectious disease outbreaks

**DOI:** 10.1371/journal.pone.0208775

**Published:** 2018-12-19

**Authors:** Elizabeth Hunter, Brian Mac Namee, John Kelleher

**Affiliations:** 1 Technological University Dublin, Dublin, Ireland; 2 University College Dublin, Dublin, Ireland; Centers for Disease Control and Prevention, UNITED STATES

## Abstract

Agent-based models are a tool that can be used to better understand the dynamics of an infectious disease outbreak. An infectious disease outbreak is influenced by many factors including vaccination or immunity levels, population density, and the age structure of the population. We hypothesize that these factors along with interactions of factors and the actions of individuals would lead to outbreaks of different size and severity even in two towns that appear similar on paper. Thus, it is necessary to implement a model that is able to capture these interactions and the actions of individuals. Using openly available data we create a data-driven agent-based model to simulate the spread of an airborne infectious disease in an Irish town. Agent-based models have been known to produce results that include the emergence of patterns and behaviours that are not directly programmed into the model. Our model is tested by simulating an outbreak of measles that occurred in Schull, Ireland in 2012. We simulate the same outbreak in 33 different towns and look at the correlations between the model results and the town characteristics (population, area, vaccination rates, age structure) to determine if the results of the model are affected by interactions of those town characteristics and the decisions on the agents in the model. As expected our results show that the outbreaks are not strongly correlated with any of the main characteristics of the towns and thus the model is most likely capturing such interactions and the agent-based model is successful in capturing the differences in the outbreaks.

## Introduction

With the emergence of new pathogens, such as SARS and MERS; the resurgence of diseases thought to be near elimination, such as measles and mumps; recent widespread epidemics of deadly diseases, such as Ebola; and the threat of pandemics from swine flu, and avian flu; it is essential to be able to model and understand the spread of an infectious disease. An accurate model can help to determine which policies and actions will have the greatest impact on reducing an outbreak, or how to best prevent an outbreak from starting and spreading. In recent years, agent-based models have proven to be useful tools for modeling, and planning, for disease outbreaks. For example, the EpiSimdemics model determined that, counter-intuitively, sequestration of military populations during an outbreak may lead to more infection [1]. More recently Olsen and Jepsen (2010) used an agent-based model to determine cost-effectiveness ratios for HPV vaccinations and determined that while a new vaccination program will incur costs, in the long term it will save on overall treatment costs and improve quality of life and survival.

In this work, we propose using an agent-based model to simulate the spread of an airborne infectious diseases in Irish towns. Furthermore, we argue that agent-based models are able to capture complex interactions between factors and emergent results based on agents’ decisions within the model that other types of models cannot. We feel that these interactions and emergent results are essential in understanding the dynamics of an outbreak. This paper presents a data-driven agent-based model to simulate infectious disease in Irish towns. To our knowledge there is no other model currently being used for the Irish context. We use only publicly available open data sources to create the model which leads to greater reproducibility. The growth of big data and more data sets becoming openly available allows for the creation of more detailed agent-based models. Governments are making data sets more widely available, allowing anyone to have access to data sets on topics such as population, health, economics and transportation. Often times the links to such data sets are being made easily accessible on one platform. For example, Ireland’s open data portal (data.gov.ie) or the city of Glasgow’s open data website (data.glasgow.gov.uk). The more realistic a model is to the society in question the easier it is interpret the results of the model and apply those results to real world scenarios. Openly available data has the additional advantage of reproducibility as anyone has access to the data to recreate the model or update the model with new data. In addition, although our model is tested on the Irish context it is easily portable between towns in Ireland and if the same level of data exists for a town in a different country our model could be used to simulate an outbreak for towns in a various countries.

The paper proceeds as follows, in the “Agent-based models for infectious disease epidemiology” section we discuss the state of the art for agent-based modelling of infections diseases. Following this the “Data” section we discuss the data sources used to create our models and in the “Model” section we detail the structure of the models. The “Model Evaluation” section present a suite of experiments that have been performed to demonstrate the effectiveness and flexibility of our models. We simulate a measles outbreak that occurred in Schull, Ireland in 2012 and show that the actual outbreak can be reproduced through agent-based simulation. To show the flexibility of our approach, in particular its ability to model many different towns, we simulate the same outbreak in 32 additional Irish towns, some of which are similar to Schull and some of which are quite different. We then perform a sensitivity analysis to understand the source of differences in the outbreaks simulated in different towns. Finally, in the “Conclusion” section we conclude the paper and suggest directions for future work.

## Agent-based models for infectious disease epidemiology

Agent-based models (ABMs) are a type of computer simulation composed of agents that can interact with each other and with an environment. An agent can be anything from an individual to an organization, or body, such as a nation state. The actions of agents are governed by a set of coded rules. At each time step an agent decides what it will do: the actions can be as simple as defining which direction an agent will move in based on some simulated perception or, the actions can be more complicated such as searching for agents with certain characteristics within a given radius and socially interacting with them [2]. ABMs can capture unexpected aggregate phenomena that result from combined individual behaviours in a model [3]. Although agent-based models have been around for some time, with one of the earliest published models appearing in 1971, it was not until the late 1990s that they began to gain popularity in the social sciences. This was mainly driven by the introduction of platforms such as Netlogo, Swarm and Repast that were designed to enable non-computer programmers to create and understand ABMs. As the platforms improve and computing power expands ABMs are being applied ever more broadly [4].

ABMs are becoming popular in infectious disease epidemiology as the models can capture the dynamics of disease spread combined with the heterogeneous mixing and social networks of agents [5]. To realistically model an outbreak, and to be useful in real world scenario, an ABM needs to model characteristics of a disease (such as infection rates), as well as characteristics of the agents and their environment, all at an appropriate level of detail [6].

One way to categorise ABMs used for infectious disease modelling is into those that use data and those that do not. It is possible to capture the dynamics of a system, such as the spread of an infectious disease, without the use of data. For example, the Dunham [7] model does not use any data to set up their population or run the model. However, for infectious disease modelling an ABM that does not use data has a disadvantage concerning applicability. While the model may be used to better understand the general dynamics of a disease, many things affect an outbreak including the characteristics of the population and the environment. It would be impossible to capture the effects these would have on the outbreak without the data to create them. Many models, such as Rakowski et al. [8] and Crooks and Hailegiorgis [9], use data sources to set up their model. Rakowski et al. [8] use both Polish census data and landscan data (https://web.ornl.gov/sci/landscan/), which is a global population distribution dataset, to create their influenza simulation. Crooks and Hailegiorgis [9] use data from a refugee camp and GIS elevation data for a model on the spread of cholera. Because both models use data the results can be directly applied to a real population and can help to influence future policy.

Infectious disease ABMs can be categorised into models that are created to simulate a specific disease or specific outbreak and those that are created to simulate general disease dynamics [6]. There are many ABMs that focus on specific strains of influenza such as H1N1 [10] or H5N1 [11], or treat influenza generally [8]. Other agent-based models have been based on specific outbreaks, for example the model by Merler et al. [12] that simulates the Ebola outbreak in Liberia. Not only does this model include specifics to how Ebola spreads, such as contact at funerals, but the model is specific to Liberia including the number of hospital beds that were used for Ebola patients over the course of the epidemic [12]. Specific ABMs have also been created to determine the effects that the government mandates had on the spread of the H1N1 virus in Mexico [10] and how vaccination programs affect the incidence rate of Human papillomavirus (HPV) in Denmark [13].

Modelling frameworks can be used to study disease dynamics, for example the models by Duan et al. [14] and Dunham [7]. These more frameworks are commonly used to influence public policy. For example, FRED (A Framework for Reconstructing Epidemiological Dynamics) is an agent-based modelling system that is used to support research on the dynamics of infectious diseases particularly for state and county public health officials to evaluate the effects of interventions [15].

## Data

In order to create the models described in this paper different types of data are needed including population statistics, GIS data, school and workplace locations and vaccination data. The majority of data used comes from Ireland’s Central Statistics Office (CSO) [16], but other sources are also used. The following sections outline the sources of the data used in the model.

### Population statistics

Population statistics are used within the model to create a realistic population of agents. Real data is used to determine the age and gender breakdowns of our populations along with correct distribution of household size and other household characteristics such as child age. The CSO provides a wealth of open access data. The data is taken from the results of the Irish census which occurs every five years. The data used for our model is from the 2011 Irish census, data from the 2016 census has recently been made available, however the 2011 data is more suitable for the outbreak we attempt to simulate as it occurred in 2012. The census data is organized into fifteen different themes each with a set of tables containing information on the population of Ireland under that theme. The themes are described in Table 1. The themes used to create the model are theme 1, theme 4, theme 5 and theme 8.

**Table 1:** The 15 themes from the CSO census data tables [17].

Theme 1: Sex, Age and Marital Status	Theme 9: Social Class and Socio-Economic Group
Theme 2: Migration, Ethnicity and Religion	Theme 10: Education
Theme 3: Irish language	Theme 11: Commuting
Theme 4: Families	Theme 12: Disability, Carers and General Health
Theme 5: Private Households	Theme 13: Occupation
Theme 6: Housing	Theme 14: Industries
Theme 7: Communal Establishments	Theme 15: PC and Internet Access
Theme 8: Principal Status	

Data can be downloaded at multiple geographic levels, the lowest being the small area [16]. Small areas are areas of population that contain between 50 and 200 dwellings. We base our simulations on data at the small area level. The CSO make available a data set (delivered in csv format) for all small areas in Ireland containing data for each table within each theme. When simulating a specific town the small areas related to that town and the necessary tables can be selected from the data set. The small area boundary file discussed in the next section provides a mapping between small areas and towns. Table 2 contains the information on the different CSO tables that were used to create the simulation.

**Table 2:** Differences in model results based on changes in the probability of infection in the model.

Infectivity Rate	*R*_0_	Average Number Infected	Percent Outbreaks	Max Infected
0.01	15	16.16	74	71
0.02	30	50.31	82	95
0.03	45	72.62	93	123
0.04	60	87.01	97	129
0.05	75	91.77	95	135
0.06	90	104.3	96	146
0.07	105	112.4	97	165
0.08	120	120.3	98	165
0.09	135	127.3	94	187
0.1	150	141.5	97	191

### GIS data

Various sources of GIS data are used in our models. GIS data not only gives us the town boundaries but also residential, commercial and recreational areas within the town that help to define where the agents live, work, and travel.

The CSO provides access to boundary files from the 2011 census. The files contain the boundaries at different levels including provinces, counties, electoral divisions, towns and small areas [16]. The data set downloaded from the CSO website contained small area information for all of Ireland: the QGIS [18] software was used to select only the small areas that overlapped with the town being simulated so the data could be loaded into Netlogo. The small area boundaries do not always match town boundaries, thus the small area data set could potentially cover more area than the town being simulated.

Zoning data is taken from two sources: Open Street Maps [19] and Myplan.ie [20]. Myplan.ie gives the shape files that include local area development plans. While Open Street Maps provides land use data. The land use data is a shape file that provides information on if the land is used for residential, commercial, retail or industrial purposes. The data set can also provide more detailed information such as if the land is used for religious purposes, sports pitches, cemeteries or reservoirs. Neither source is comprehensive and there are some areas in the towns for which zoning data is not available. The different zoning and land use types are sorted into six categories: open, town center, community, residential, commercial and mixed.

### School locations

In order to determine both the number of schools in a town and their locations we use data from the Department of Education and Skills in Ireland. They provide data on individual schools, including enrollment and type of school (primary or secondary) [21]. The data set includes the longitude and latitude of the schools. These are then geocoded in QGIS [18] in order to create a GIS shape file that can be combined with the town boundary and land use shape files and loaded into Netlogo.

### Vaccination data

Vaccination statistics are used to determine the number of agents in our model who have been vaccinated and thus are immune to the disease. Vaccination statistics for Ireland can be found on the childhood vaccination schedule. Statistics are presented for Ireland as a whole and broken up into Health Service Executive (HSE) regions. Vaccination uptake statistics for the whole of Ireland and by HSE region are available on Ireland’s Health Protection Surveillance centre website going back to 1999 [22]. The Organisation for Economic Co-operation and Development (OECD) reports vaccination rates for Ireland back to 1983 [23]. When initiating the model the choice of all Ireland vaccination rates or vaccination rates for a specific HSE region based on the town being modelled must be made. Further discussion of how vaccination rates are used to in the model can be found in the “Society” section.

## Model

We use the computer software Netlogo [24] to implement the simulations described in this paper. However, the open-data driven approach described in this paper could be used with any agent-based modelling tool or platform. Netlogo is an easy to use and popular environment for creating ABMs [4]. It does, however, have disadvantages, one of the biggest being the speed of the program. When modelling simulations with a small number of agents Netlogo works well, however, once the number of agents gets large the simulation can become prohibitively slow.

The model used in the experiments described in this paper can stimulate outbreaks of measles-like diseases in small to medium sized Irish towns. Each agent in the model simulates the behaviour of a resident of the town. The simulations of the towns and the behaviours of the agents in the towns are driven by the data sets described in the previous section. The model we use in the experiments is outlined using the ODD protocol [25] in the Appendix 1 of the paper and can be found online in the Netlogo User Community Model Library (http://ccl.northwestern.edu/netlogo/models/community/town_model_burnin) The model can be adapted to simulate any town in Ireland or elsewhere as long as the data sets listed in the “Data” section are available for the new towns. The following sections give a brief overview of the environment, society, transportation and disease components of the model, as well as the schedule used to simulate agent behaviour.

### Environment

**Fig 1:**
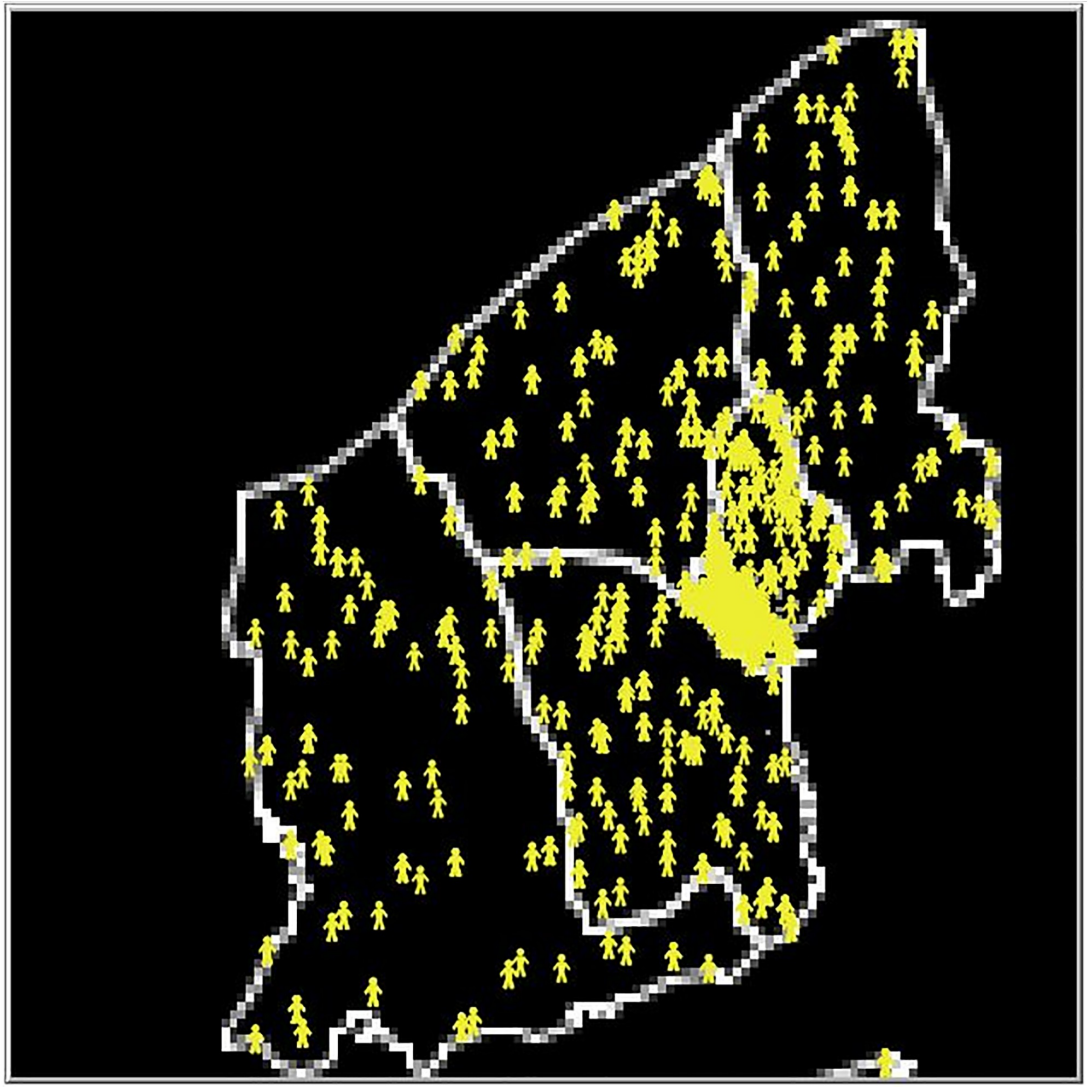
Example of model environment. The environment created by the model for the town of Schull, Ireland. The white lines are the boundaries of the small areas and the yellow agents are located at the agent households in the model.

The towns are created in Netlogo. The Netlogo world is a two dimensional grid where the squares that make up the grid are referred to as patches. For the purpose of our model each patch has an area approximately equal to 111 *m*^2^. If two agents occupy the same patch they are considered to be in the same place and in contact with each other. Small area data sets from Ireland’s Central Statistics Office (CSO) are used to create the basic geographic layout of the town. Primary and secondary schools are added into the model using data from Ireland’s Department of Education and Skills. Zoning data, as described in the previous section, is used to place households and work places in appropriate locations within the small areas and to determine community locations. The population data for each small area specifies the number of households in that small area, and a corresponding number of households is added to each small area in the simulation. The population data specifies the distribution of household types in each small area *(single, couple, couple plus others, couple with children, couple with children plus others, single parent, singles parent plus others or other)* and the houses in our simulated small areas are assigned types randomly selected following this distribution. Household types are assigned concurrently with creating agents so that accurate household structures are created. Fig 1 provides an example of the model environment created for the town of Schull, Ireland. More detail on the creation of households can be found in the “Initialization” section of the Appendix 1 “Model Description”. The model is given a start week, which is used to determine if summer holidays occur over the lifetime of the model, and a year which is used to determine vaccination rates.

### Society

Agents are added to the town based on the population data described in the “Population Statistics” section, adults are added first and given an age and sex based on the distributions of age and sex in the appropriate small area from the census data. Children are then added into households that have a type that includes children and are given an age and sex based on the appropriate distributions from the census data. All agents are given an economic status *(student, retired, looking for first job, unemployed, sick/disabled, stay at home, work)* based on the distribution in the relevant small area in the census data. Agents with the economic status of work are assigned to a random workplace within the town and agents with the economic status of student are assigned randomly to one of the schools of the appropriate level (*preschool*, *primary*, *secondary*) in the town. Irish vaccination data is used to determine the percentage of each age group that have received vaccinations for the infectious disease being modelled. For example, if 90% of 1 year olds in Ireland had been given the MMR vaccination in 2011 and we are running a model for 2012, we give each agent in the model with an age of 2 a 90% chance of having been vaccinated. If an agent is vaccinated they are given a 97% chance of being immune to the disease. This takes into account vaccination failure and is based on the vaccine effectiveness rate for measles [26]. Half of the agents with age less than 1 are given immunity to a disease to mimic passive immunity infants receive from their mothers [27]. For any agents that have an age corresponding to a vaccination year not in our data we give a 99% chance of being immune. Prior to vaccination campaigns the majority of the population would have either had or been exposed to childhood diseases, such as measles, leaving them immune in later life.

### Disease

In this work we study measles-like diseases and have designed a disease model to reflect this. Measles is a highly infectious virus that is often associated with childhood. It is most common among children between the ages of 1 and 4 years old but anyone who is not immune from previous illness or vaccination is susceptible [28]. Once a person is exposed to measles the disease has a 10-14 day incubation period before the onset of the first symptoms. Fourteen days after initial exposure, the measles rash occurs [26]. An individual can be infectious between two and four days before the onset of the rash and five days after the onset of the rash [28].

To simulate the transmission dynamics of measles in the model we use a compartmental SEIR type model. The SEIR model categorizes agents into susceptible, exposed, infected or recovered statuses and looks at movement of agents between categories [29]. Disease transmission between agents in the model occurs as follows. When an infectious agent comes into contact with an agent who is susceptible, the susceptible agent will become exposed if a random number drawn between 0 and 1 is less than the probability of infection for the disease. In the model we consider any agents who are occupying the same patch as in contact with each other. Once an agent moves to the exposed state they are assigned an exposure time which corresponds to the length of time where they will remain exposed before becoming infectious. On average people stay exposed but not infectious to measles for 10 days [26]. Agents are assigned an exposure time sampled from a normal distribution with mean of 10 and standard deviation of 0.5. After this time has passed the agent will become infectious. Again the agent is assigned a length of time where they will remain infectious before recovering, on average this is 8 days [26]. This value is sampled from a normal distribution with mean of 8 and a standard deviation of 0.5.

The probability of infection is determined using the values for *R*_0_ for measles (12-18) [26]. *R*_0_ is the basic reproductive number and is defined as the expected number of individuals infected by one infectious individual in a completely susceptible population. It is the standard measure of transmission potential of a disease [30]. Other factors such as the transmission rate or the infectivity rate of a disease are less frequently estimated than *R*_0_. *R*_0_ is typically calculated for outbreaks as it is thought of as one of the most important parameters when trying to control an outbreak [31]. Data from real outbreaks can be use to estimate *R*_0_ [32]. However, the infectivity rate requires more information and detailed contact tracing to determine the proportion of susceptible contacts an infectious individual would infect [31]. Therefore, *R*_0_ is the most commonly found parameter for disease dynamics and what we use in the model. The parameter can be broken down into three components, number of contacts per unit time (*c*), the transmission probability per contact (*p*), and the duration of the infectiousness (*d*). The relationship can be seen in Eq 1 [30].

$$\begin{array}{c} R_{0}=cpd \end{array}$$ (1)

Of the four variables in the equation, *R*_0_, *c*, *p*, and *d*, three are known or can be estimated from our model. *R*_0_ is known to be between 12 and 18 [26], *d* is known to be approximately 8 days or 96 time steps in our model, and we can determine c, the average number of contacts per agent per tick by running the model. Here we take *c* as an average across all agents, however, within the model agents movements determine their individual contact rate, thus each agent could have a different number of contacts. This number of contacts will influence the transmission of the disease or likelihood of infection, if an agent has a large number of contacts they should have a higher chance of becoming infected and will spread the disease to more agents. The model for each town is run 20 times with no infection. For each tick the agents keep track of the number of other agents they have come into contact with. This number is averaged across agents at the end of the model run and then the average is taken across the 20 runs. Once the average contacts per time step is calculated the probability of infection is calculated by rearranging the *R*_0_ formula as follows:

$$\begin{array}{c} p=\frac{R_{0}}{cd} \end{array}$$ (2)

The model was tested using values for *R*_0_ within the range of 12-18 and the value of 12 was selected as it the result from a model with an *R*_0_ of 12 seemed to best match the results of the 2012 measles outbreak in Schull, Ireland that’s discussed later in the “Case Study: Schull 2012” section.

Although we present the model using measles as the infectious disease it is possible to adjust the model for any airborne infectious disease where transmission is determined by SEIR dynamics, such as influenza or mumps.

### Transportation

In the model agents use straight-line transportation. Agents will move between their home and destination in a straight line following the most direct route. Agents move in steps from one adjacent patch to the next and will reach their destination within one time step. Although this is a naive transportation model, for small towns, such as those described in the paper, where distances travelled are short it is a reasonable simplification.

### Schedule

The model is run in Netlogo using discrete time steps and runs from initialization to the point where there are no more agents who are exposed or infected. The behaviour of each agent is updated at each time step. The time steps represent two hours in a day, with 12 steps representing one day. Each time step an agent moves throughout the town. During a week day agents leave their home in the morning and go to school or work. At the end of the school/work day agents return home. Agents who are not students or working will move randomly throughout the town, choosing different locations within the community to move to during daytime hours. On weekend days all agents move randomly throughout the town during the day and during summer weeks students will move randomly throughout the town every day. Further discussion of the movement of agents can be found in the “Submodels” section of the Appendix 1 “Model Description”.

## Model evaluation

The following sections describe the evaluation and testing of the model. First we present results from tests to illustrate that the model is working as expected for a measles outbreak. We will analyse the course of simulated outbreaks, by examining the infection curves created by the model, and then we run a sensitivity analysis looking at three parameters: the probability of infection, the vaccination rate, and the chance that a student will stay home sick and self isolate. The infection curve will help us to determine if the model is following the expected pattern for an SEIR model. The sensitivity analysis will help to determine if the model acts as expected when parameters in the model are adjusted. For example, as vaccination rates, and thus the number of immune agents, increase the resulting size of the outbreak, and the chance that an outbreak will occur, should decrease. Similarly, if agents have a higher probability of staying home when infected, they should infect fewer agents and the outbreak should be less severe. To further test our model we run two additional experiments. The first is to show that the model is capable of recreating a historical outbreak, specifically a case study simulating a measles outbreak that occurred in Schull, Ireland in 2012. We then show how easy it is to simulate a similar outbreak in a host of towns, and, finally compare the results from the various towns against the historical outbreaks. By showing that there are no obvious correlations with the model results and town characteristics we show that the model captures interactions between these characteristics and agents actions.

### Modelling disease dynamics

**Fig 2:**
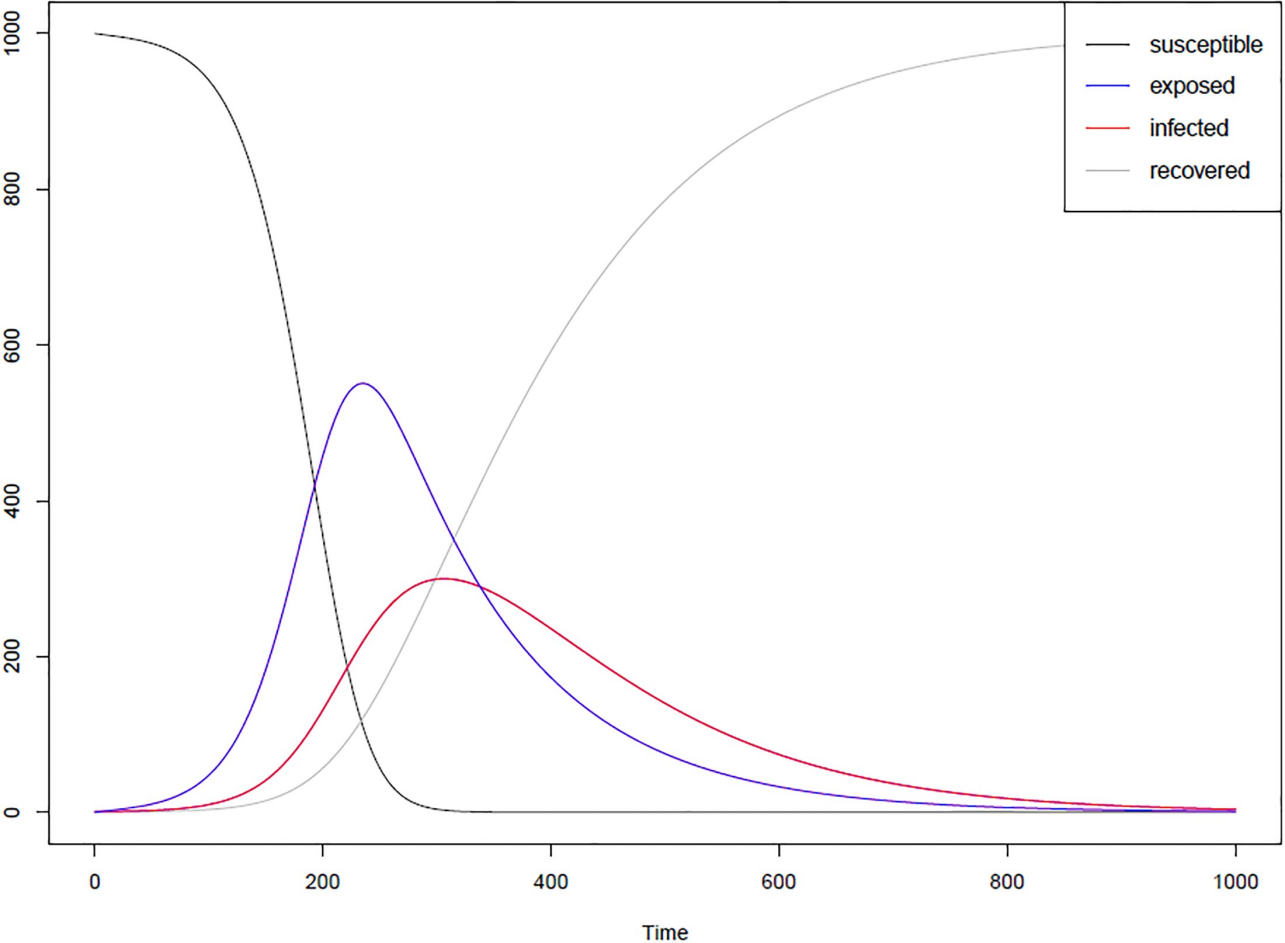
Example SEIR curves. The curves are generated using a basic SEIR differential equation model. Equation based models have been proved to produces accurate results for real epidemics [33].

**Fig 3:**
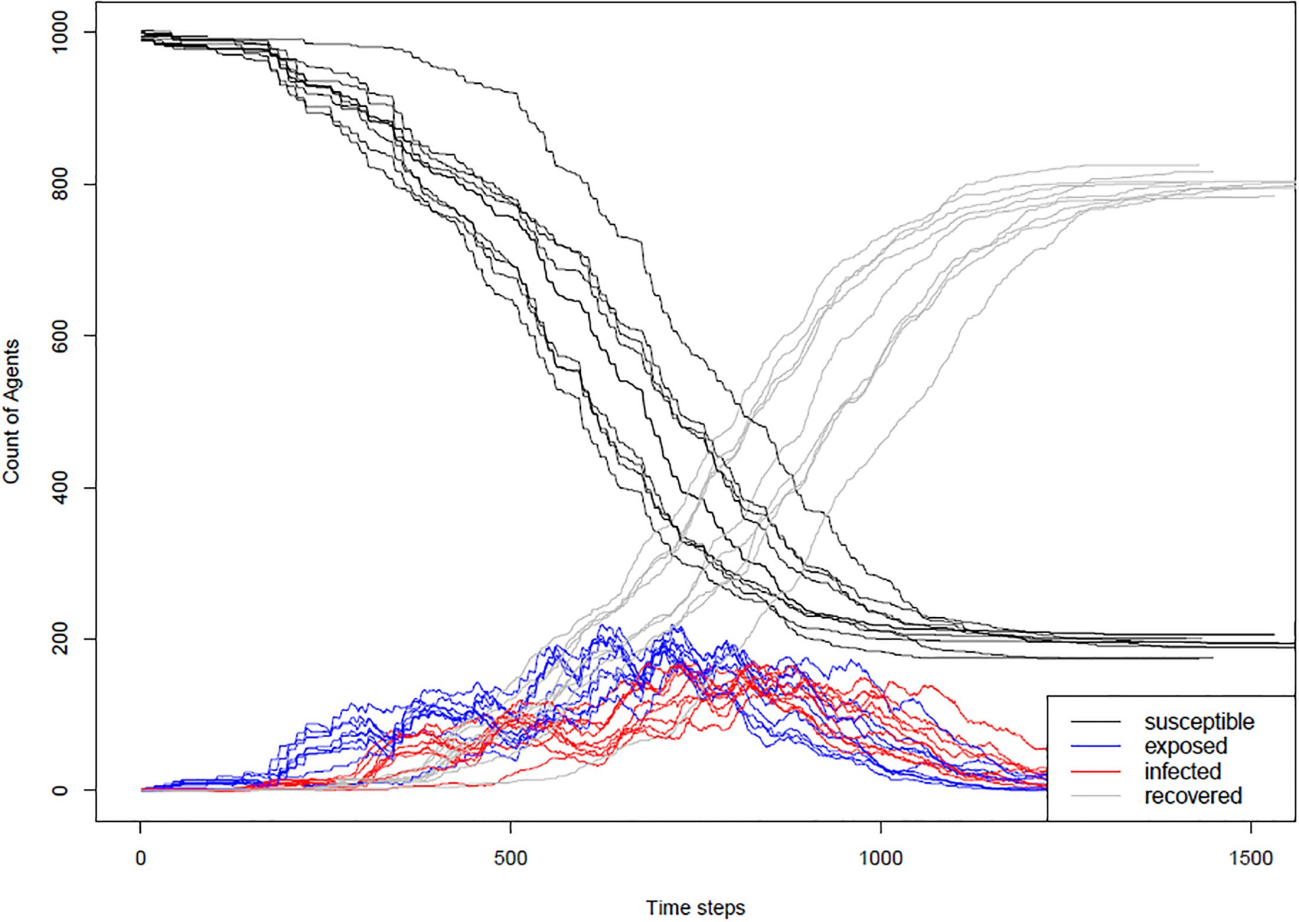
SEIR curves from the model. SEIR infection curve for 10 runs of the model in a town in which no one was vaccinated or immune.

When modelling an infectious disease, one important test is to determine if the model is capturing the correct disease dynamics. The dynamics of an SEIR model should roughly follow the curves in Fig 2. The SEIR infection curve plots the number of agents in each of the four categories—susceptible, exposed, infected and recovered at each time step. In order to test that our model will produce these curves, the model was run ten times for a town (Schull, Ireland) under the case where no one in the town was vaccinated or immune and Fig 3 shows the results of these runs in the form of the SEIR infection curve. The curves from the model match the shape of the classic SEIR curve. Fig 3 includes the curves for all 10 runs to illustrate that while the overall pattern of the outbreak remains the same on each run, the stochastic nature of the model means that in some runs the outbreak takes off more quickly than others. We also looked at where infections took place in the model and determined that for the town of Schull, across 100 runs, 96.5% of infections occurred in a school setting, 3% occurred at home and less than 1% occurred in work and community settings.

### Sensitivity analysis

After checking the model to make sure it produced the expected disease dynamics, a sensitivity analysis was run on several parameters. The sensitivity analysis was run to determine if the model responds as expected to changes in different parameters. The parameters for infectivity of the disease, vaccination rates and the percent of times an agent stays home when sick were investigated. For all analysis the models were run on the town of Schull, Ireland, and unless otherwise noted use the all Ireland vaccination rates. For each analyses we look at some or all of the following characteristics of the outbreak to determine how it changes: average number of agents infected, percent of outbreaks that occur and the maximum number of infected agents across runs. For the percent of outbreaks that occur we use the World Health Organization’s definition of a measles outbreak to determine when an outbreak has occurred. The World Health Organization’s considers a measles outbreak to be two or more cases of measles that are temporally related, and epidemiologically or virologically linked or both. In the model we consider an outbreak to be at least one agent infected by the initially infected agent.

#### Sensitivity analysis: Infectivity

**Fig 4:**
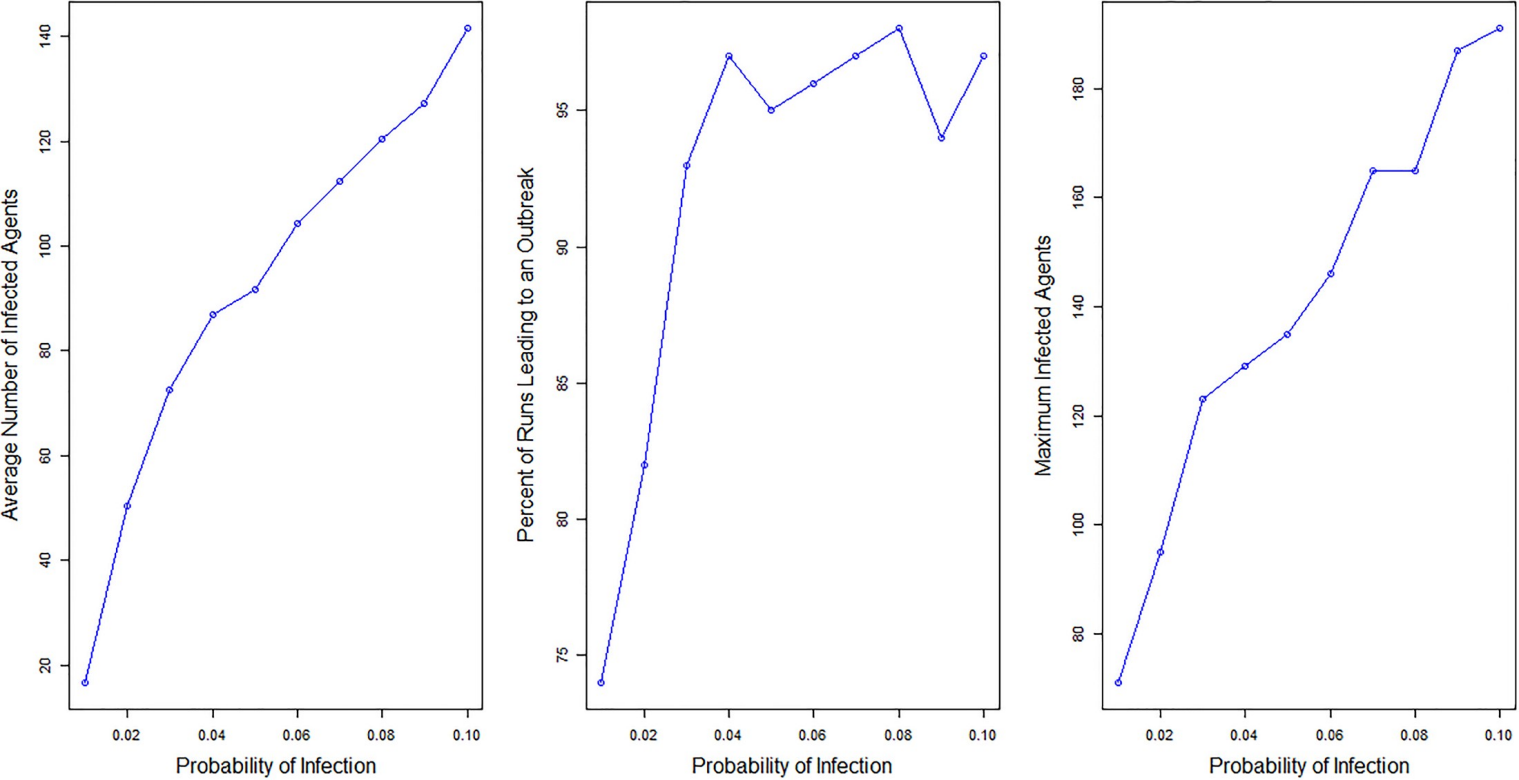
Outbreaks by probability of infection. Charts showing the change in average number of agents infected, the percent of runs leading to outbreaks and the maximum infected agents as the probability of infection changes.

To look at how changes in the probability of infection influence the resulting outbreaks we run the model 100 times each for ten different probabilities. The probabilities of infections we use range from 0.01 to 0.1, the *R*_0_ values that correspond with the probabilities of infection range from 15 to 150. These *R*_0_ values are highly unlikely to occur in a real scenario, however, the probabilities of infection for the sensitivity analysis were not chosen because of their realistic values but instead as a method of testing the model to determine if it behaved as expected. All other parameters remain constant, including the town, which is Schull, the vaccination rates, and the home sick parameter, which is set to a 70% chance of staying home when sick. If the disease transmission is working correctly in the model there should be an increase in the size and severity of outbreaks as the probability is increased. Table 2 and Fig 4 show the results for the ten different probabilities of infection. Analysis of the results show that as the probability of infection increases, meaning that an infected agent has a higher chance of passing the virus on per contact, the average number of infected individuals, and the maximum number of infected agents all increase. There are a few times when the percent of runs resulting in an outbreak decreases by a few percentage points (from probability 0.04 to 0.05 and from 0.08 to 0.09). This result is not concerning as in both cases the decrease is only by a few percentage points and is not surprising given the stochastic elements within the simulations.

#### Sensitivity analysis: Vaccination rates

Three alternative vaccination scenarios were considered to determine if the disease dynamics respond as expected to changes in vaccination rates. The vaccination scenarios used were: no vaccinations and no immunity, full vaccination and full immunity, and vaccination to the herd immunity level. All other parameters remain constant, including the town which is Schull, the probability of infection which is set to 0.008, and the home sick parameter which is set to a 70% chance of staying home when sick. If the model is working as we expect, in the full vaccination scenario we expect the outbreak should either be very small or not occur at all, in the full vaccination scenario we expect that an outbreak should not take off and in the herd immunity scenario we expect that any outbreaks that do occur should be small.

Herd immunity is the concept that there is a critical number of individuals that need to be vaccinated or immunized to interrupt the transmission of an infectious disease in a population. The formula to determine the proportion of individuals who need to have been vaccinated to achieve herd immunity is:

$$\begin{array}{c} p=(1-\frac{1}{R_{0}}) \end{array}$$ (3)

However, as the vaccinations are not always successful, the proportion needs to be adjusted by the vaccine effectiveness in order to find the critical vaccination coverage needed to reach herd immunity. The equation for the critical vaccination coverage is

$$\begin{array}{c} V_{c}=\frac{\left(1-\frac{1}{R_{0}}\right)}{V_{e}} \end{array}$$ (4)

For measles we take *R*_0_ as 12 and *V*_*e*_ as 97% [26], which gives a critical vaccination coverage level of 94.5%. To reach the herd immunity within our population, we determine the percent of immune agents (agents vaccinated in the model, given immunity due to an age that would suggest they had measles as a child, or given immunity from maternal antibodies as discussed in the “Society” section) in the population, and then calculate the remaining number of agents who need to be vaccinated, in order for 94.5% of the population to be immune.

The model was run 100 times for each scenario. Results from all three scenarios are shown in Table 3. The average number of infected individuals across runs, the percent of outbreaks across runs and the maximum number of infected agents in any runs are compared. The main results of the analysis can be summarised as follows:

- With no agents in the town immune, outbreaks occur in 94% of runs. The average number of infected agents across the runs is 726 and the maximum number of infected agents in any run is 846. This is as expected as with no agents immune the virus can spread quickly through the town unimpeded.
- As expected with full vaccination and immunity, outbreaks do not take off. The initial infected agent does not infect any additional agents in any run.
- In the herd immunity scenario, 52% of the runs results in outbreaks, however, the average number of infected individuals across the outbreaks is 3.28 and the maximum number of agents infected in any run is 15. The lower average infected and maximum infected numbers provide evidence that when our population reaches herd immunity the likelihood of an outbreak is significantly reduced and the size of the outbreak is also reduced.

**Table 3:** Vaccination scenarios sensitivity analysis.

Immunity Level	Average Infected	Percent Outbreaks	Max Infected
No Immunity	726.7	94	846
All Immune	1.0	0	1
Herd Immunity	3.3	52	15

#### Sensitivity analysis: Home sick parameter

**Fig 5:**
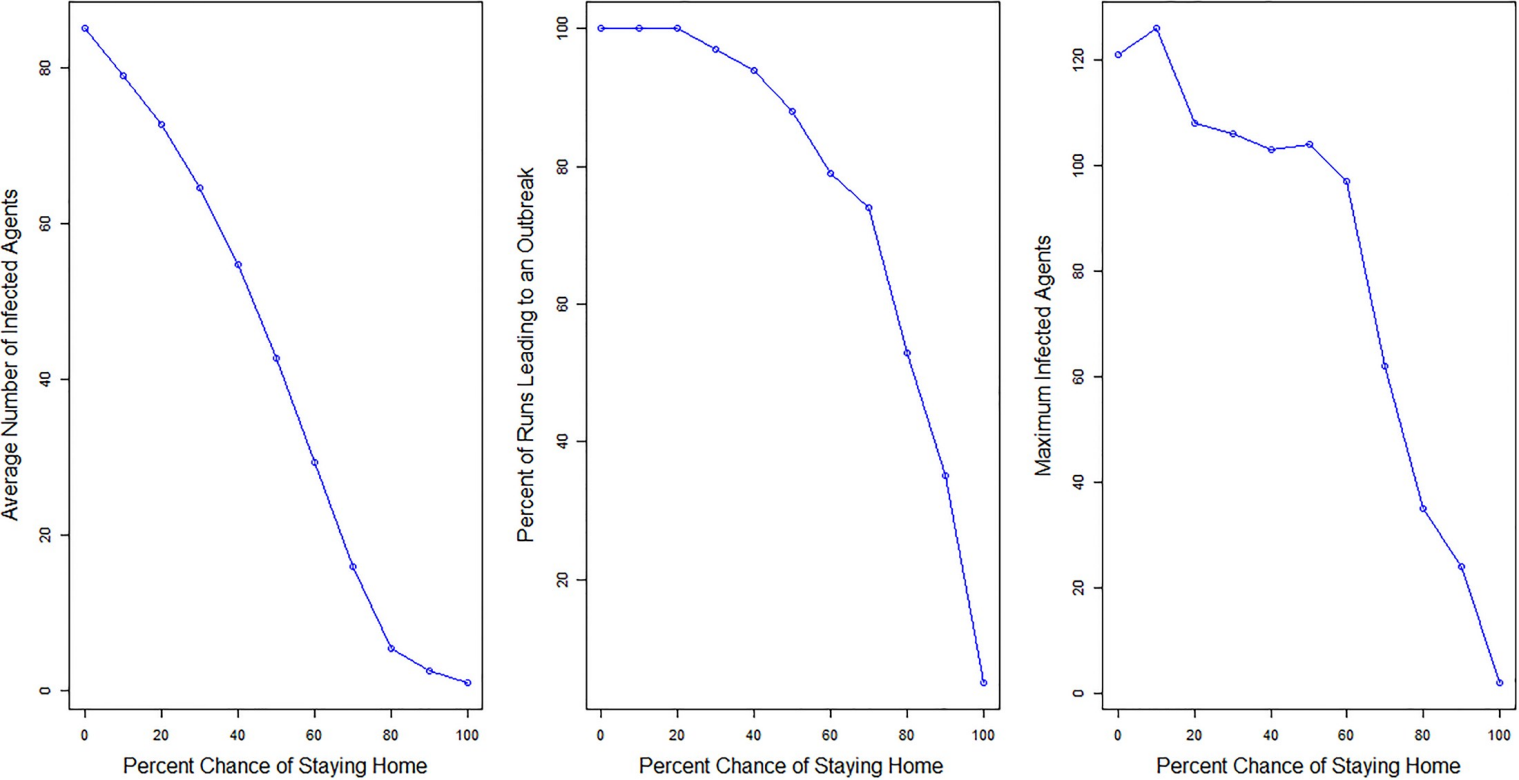
Outbreaks by percent chance of staying home sick. Charts showing the change in average number of agents infected, the percent of runs leading to outbreaks and the maximum infected agents as the percent chance of staying home sick increases.

The parameter to determine the percent of time an agent stays home when sick is also adjusted as another method to determine if the model is working as expected. If agents stay home more frequently when infected they will be less likely to interact with other agents and pass the infection on to others. Therefore, it is expected that if agents are more likely to stay home then the outbreak will be less severe while if an agent is less likely to stay home when infected the outbreak will be more severe. Eleven different scenarios are considered for the home sick parameter: 0, 10, 20, 30, 40, 50, 60, 70, 80, 90, and 100%. All other parameters remain constant, including the town which is Schull, the vaccination rates, and the probability of infection which is set to 0.008. The model is run 100 times for each new parameter value. Results can be found in Table 4 and Fig 5:

**Table 4:** Differences in model results based on the percent chance of agents staying home when sick.

Chance of Staying Home	Average Number Infected	Percent Outbreaks	Max Infected
100%	1.0	5	2
90	2.6	35	24
80	5.4	53	35
70	15.9	74	62
60	29.3	79	97
50	42.7	88	104
40	54.8	94	103
30	64.6	97	106
20	72.8	100	108
10	79.1	100	126
0	85.1	100	121

As can be seen from Table 4 and Fig 5, as the percent chance of staying home decreases the average number of infected agents and the percent of runs that lead to an outbreak increase. This is further evidence that the model is working correctly as this is what is expected.

### Case study: Schull 2012

To evaluate the ability of our model to replicate an actual outbreak of measles we present a case study that focuses on simulating a real measles outbreak that occurred in Schull, Ireland in 2012 [34].

As an agent-based model is designed to be stochastic in order to reflect the different decisions that individuals make in the real world, we do not expect that our agent-based model will identically reproduce the Schull outbreak every time it is run. In fact, we do not necessarily expect to see a perfect replication of the outbreak for a majority of runs. The Schull outbreak happened as it did because of decisions made by infected individuals: for example a decision by a person to go to school while feeling ill would most likely increase the number of infections in the outbreak, while a decision by the same person to stay home would most likely have the opposite effect. Our agent-based model captures these different scenarios as the agents decide at each time step in the simulation what their actions are going to be.

Instead of showing that the model perfectly replicates the Schull outbreak, we want to show that the Schull outbreak falls within the range of outbreaks that our model predicts. We will show this by looking at the size of the outbreak, the ages of agents infected, the number of agents infected per week and the length of the outbreak. The following sections discuss the Schull outbreak in more detail and present the results from our case study.

#### Schull measles outbreak

Schull is a town in county Cork, Ireland. The town has a population of approximately 1,000 individuals and spans an area of about 17.1 *km*^2^. Schull suffered a measles outbreak in the early Summer of 2012. Over a three-month period starting on 9 April 2012 and ending 15 June 2012 there were 63 cases of measles notified in West Cork (the region of Ireland in which Schull is situated in) [34]. The initial focus of the outbreak was on the Schull and Skibbereen areas but as the outbreak went on cases were notified throughout West Cork including Bantry, Bandon, Dunmanway and Clonakilty [35]. Seventy-eight percent of the cases were in the 10-19 year age group, and only four cases were in the under-five age group. Lack of vaccinations is thought to have played a large part in the outbreak. Vaccination status is known for 59 of the 63 cases: out of those 59 cases 55 were unvaccinated [34]. According to the Health Services Executive in Ireland, at the time of the outbreak the uptake of the MMR vaccine at age two years was 92% nationally, however, in West Cork the uptake was 86%: West Cork has historically been an area of low uptake of vaccinations. In response to the outbreak, it was recommended that infants aged 6-12 months resident in the area have an early dose of the MMR vaccine, and unvaccinated siblings of infected individuals were asked to self-isolate for the duration of the incubation period. Of the 63 cases in the West Cork region approximately 30 cases were in Schull. [35].

#### Schull model and parameters

In order to create a model that accurately represents the Schull outbreak, parameters need to be adjusted for the town. We select a start week of 15 which corresponds to the timing of the Schull measles outbreak starting in the 15th week of the year. We also set the probability of infection to 0.008 which is derived using Eq 1. West Cork vaccination rates for MMR were used and the initial infected individual is set up as a student who was not immunized. The model was run 400 times with the Schull parameters.

#### Schull results

The stocasticity in the simulation leads to different results each time the model is run. The average number of agents infected across the runs was 17 with a maximum of 90 infected agents in one run. Twenty-five percent of the runs results in outbreaks that had more than 30 agents infected.

**Fig 6:**
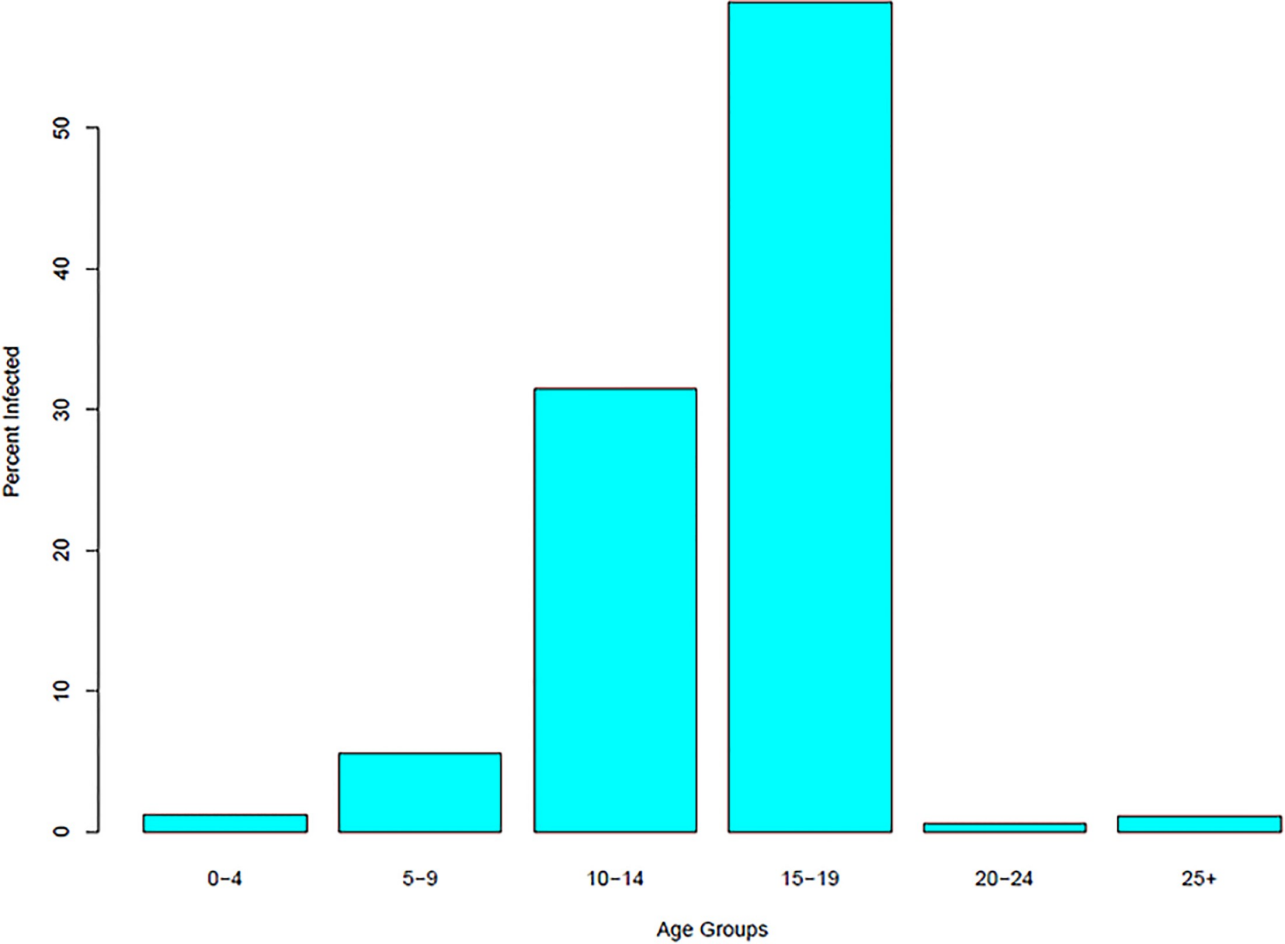
Average number of agents infected in Schull by age groups.

The results show that while the average for all the runs is lower than the number of infected in the Schull outbreak, the number of people actually infected is in the 75th percentile of model runs. The Schull outbreak was primarily made up of individuals between the age of 10-19. The average percent of infected agents in each age group is shown in Fig 6.

The distribution of infected agents by age matches what occurred in the Schull outbreak with the majority of individuals being infected in the age groups between 10 and 19. This is taken as evidence that the model simulates a scenario similar to what actually happened.

**Fig 7:**
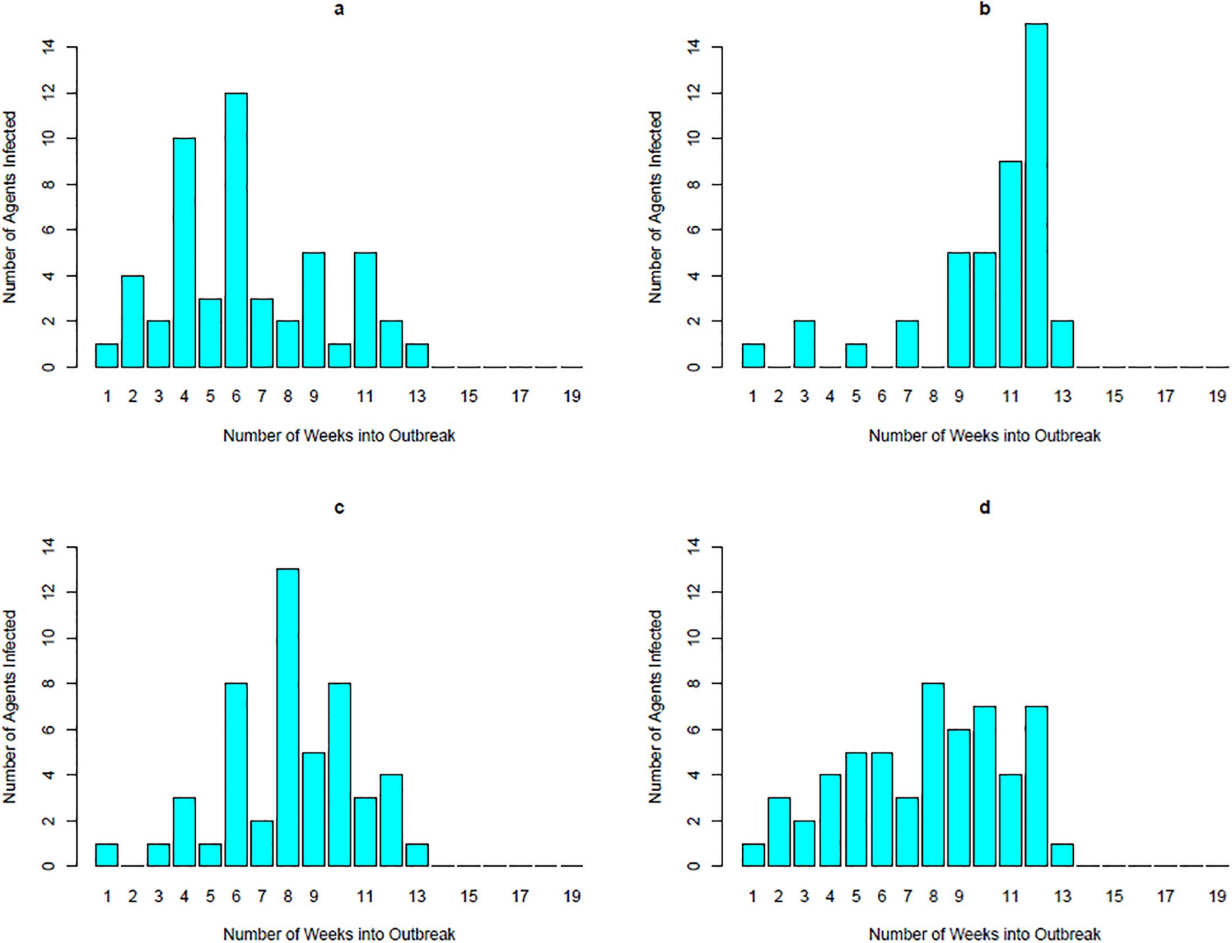
Distribution of agents infected by week in four different runs.

The outbreak patterns are also analysed to determine if our model results in outbreaks that follow the same path as the actual outbreak. The reported cases for the Schull outbreak peak 3 and 5 weeks after the initial case is reported. Some of the runs match closely with the outbreak pattern for Schull and some do not. Fig 7 shows some of the different outbreak patterns that occur in our model. Fig 7*a* and 7*b* are most similar to the Schull outbreak with peaks of cases two weeks apart.

Although our model does not predict perfectly what happened in Schull every run, we believe that a number of the runs do capture the outbreak scenario and that the Schull outbreak falls within the range of outbreaks in our simulation. The fact that the model produces 25% of runs with the number of agents infected similar to the Schull outbreak, the age distribution of the agents infected in the runs and that a number of runs match the distribution of cases by week found in the Schull outbreak are taken as evidence that we are capturing an outbreak similar to the one that occurred in Schull.

### Simulating additional towns

The main advantages of agent-based models is their ability to capture heterogenity along with complex behaviours, interactions, and characteristics of agents. We add to this the ability to accurately simulate different towns based on data from publicly available data sources. We demonstrate this by simulating outbreaks similar to what happened in Schull in 32 other Irish towns. We show that subtle differences between these towns mean that the outbreaks follow different profiles to that seen in Schull. We pick a subset of these towns that are similar to Schull in terms of population, area, or both for deeper analysis. The following sections discuss simulating a measles outbreak on 33 different towns including Schull and the differences in those outbreaks. We then choose towns that are similar in population but not area, area but not population, or both to Schull and how the outbreaks from the simulation compare to the Schull simulation.

The towns selected are included in Table 5 which also describes their key characteristics. For each of the towns we simulate a measles outbreak with the same conditions as Schull. To limit the variability in the models to only characteristics of each town such as area, population size, age structure and number of schools, the vaccination rates for all of Ireland are used instead of region specific rates. Schull is rerun with the all Ireland vaccination rates in order to make the results comparable to the other towns. To calculate the transmission probability for each town we find the average contacts per time step and then use the formulas in the “Disease” section. Table 5 has a column including the transmission probabilities for each town. All other model parameters are the same as in the Schull model from the previous section. For each town the model is run 200 times and the percent of runs where an outbreak occurs (the initially infected agent passed the virus to at least one other agent) is determined. The proportion of runs with outbreaks (two or more cases of measles) for each town is listed in the rightmost column in Table 5. We can see that the number of runs that result in an outbreak in each town are quite different. Even in some cases where the size and populations of the towns are similar (e.g. Bagenalstown and Ballyjamesduff) we see quite different outcomes. This is evidence of the value of agent-based modelling and shows the value in being able to simulate towns accurately based on publicly available data sources.

**Table 5:** Area, population and other characteristics for each of the 33 selected towns.

Town	Population	Area(*km*^2^)	Probability of Infection	Small Areas	Students	Not Immune	Density	Outbreak	*R*_*e*_
Arainn	1,251	47.48	0.009	6	30.7	14.7	26.34	69.5	1.76
Ardamine	3,293	23.33	0.006	19	33.8	12.4	130.00	88.5	1.49
Ardfert	997	7.97	0.006	4	38.5	13.4	125.09	65.0	1.61
Arranmore	514	18.08	0.055	4	31.3	8.4	28.43	69.5	1.01
Bagenalstown	3,421	18.00	0.003	13	35.2	12.8	190.06	66.0	1.54
Ballyjamesduff	3,134	21.60	0.005	12	37.4	13.5	145.09	83.5	1.62
Banagher	1,993	19.85	0.009	8	36.5	12.7	100.40	88.5	1.52
Blarney	5,310	23.30	0.001	21	36.9	13.2	227.90	60.0	1.58
Castlereagh	3,077	40.09	0.013	15	26.3	10.2	76.75	85.5	1.22
Clane	7,527	18.89	0.002	28	36.5	14.5	398.46	86.0	1.74
Croom	1,690	18.17	0.004	6	35.7	11.4	93.01	57.0	1.37
Donegal	4,010	31.49	0.006	17	30.4	12.5	127.34	92.0	1.5
Gort	2,671	11.21	0.009	12	27.6	11.6	238.27	87.5	1.39
Kenmare	2,912	55.61	0.006	17	29.4	10.3	52.36	81.5	1.24
Kilcock	6,234	16.40	0.001	23	35.7	14.4	380.12	58.0	1.73
Kildare	9,325	37.09	0.002	32	36.1	14.0	251.42	88.5	1.68
Kilkee	1,037	5.26	0.008	8	25.7	9.1	187.15	65.5	1.10
Killadysert	922	63.96	0.009	4	36.1	10.0	14.52	61.0	1.20
Kinsale	6,871	12.96	0.003	31	30.4	11.5	129.03	87.0	1.38
Lisdoonvarna	861	12.96	0.010	3	26.5	12.3	66.44	63.0	1.48
Louisburgh	983	23.30	0.009	7	27.6	11.8	42.19	62.5	1.42
Moate	3,046	21.34	0.007	12	32.6	12.7	142.74	90.0	1.52
Oranmore	4,325	22.38	0.002	18	28.9	13.1	193.25	62.0	1.57
Portmagee	390	16.77	0.023	2	27.7	10.3	23.26	61.5	1.24
Rathnew	3,294	6.90	0.003	10	37.4	15.0	477.39	73.0	1.80
Roscrea	6,318	48.45	0.006	26	33.2	11.5	130.40	91.0	1.38
Rosslare	2,057	17.90	0.003	12	26.0	9.1	114.92	47.0	1.10
Roundstone	459	28.01	0.041	4	31.1	11.1	16.39	86.0	1.33
Schull	987	17.03	0.008	7	30.6	11.4	57.96	72.5	1.37
Shanagolden	946	17.79	0.008	4	33.0	11.0	53.18	54.5	1.32
Stamullin	4,694	37.68	0.003	14	37.0	12.9	124.58	78.5	1.55
Strokestown	1,003	18.11	0.009	6	31.4	11.1	55.38	73.5	1.33
Tramore	9,548	16.60	0.001	36	35.8	12.2	575.18	73.0	1.46

The towns selected are included in Table 5 along with a set of factors that help to define each town and the percent of model runs that lead to an outbreak for each town. The factors are number of small areas in the town, the percent of students in the town, the percent of unvaccinated individuals in the town, and the population density.

**Fig 8:**
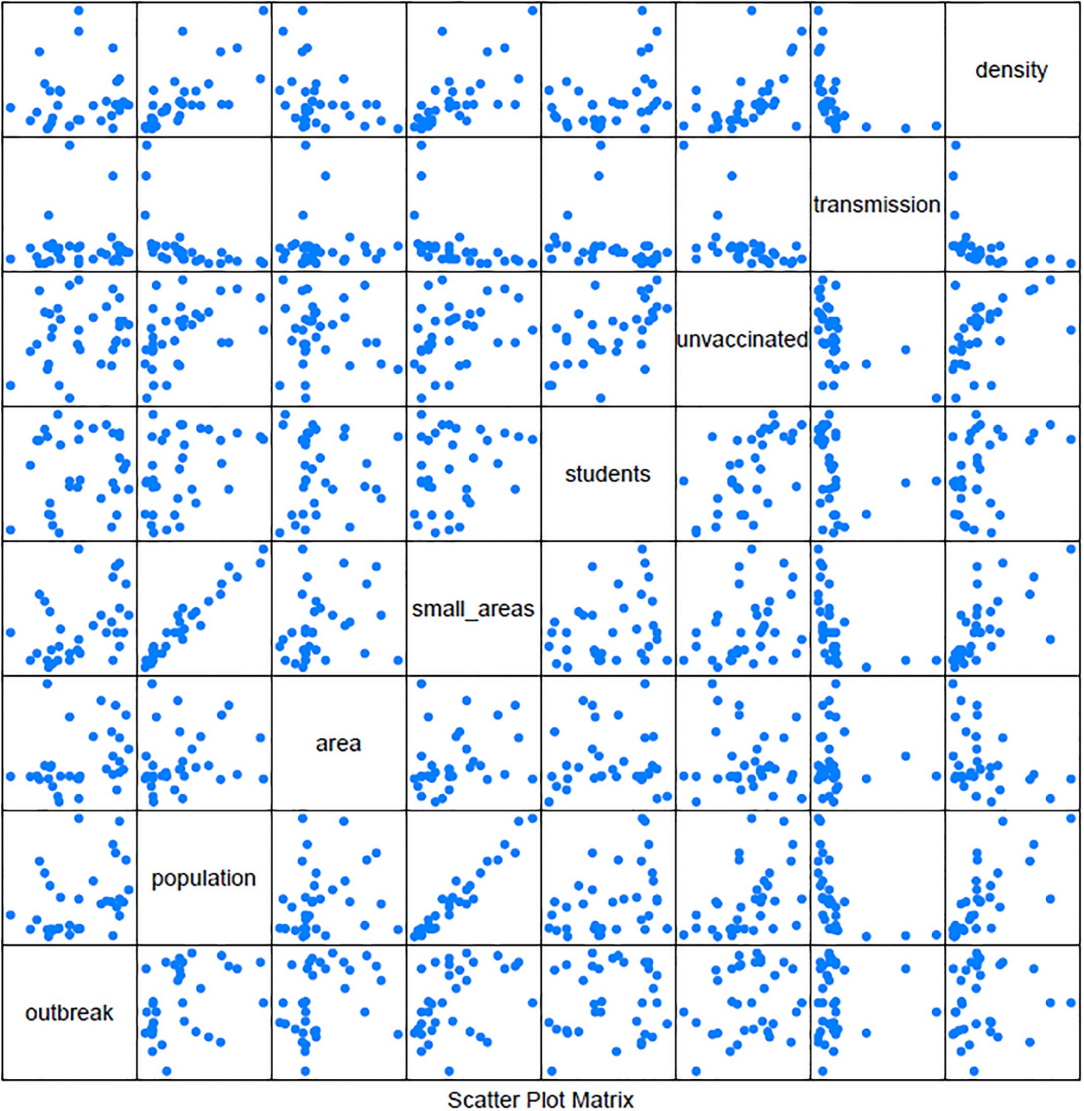
Percent of runs leading to an outbreak. Scatter plot of percent of runs resulting in outbreak and factors defining each town. *Outbreak* is percent of runs resulting in outbreaks, *smallarea* is the number of small areas in the town, *students* is the percent of students in the town, *unvaccinated* is the percent of unvaccinated agents in the town, *density* is the population density, and *transmission* is the probability of transmission per contact.

To get a better understanding of the relationship between factors and outbreaks, Fig 8 shows a scatter plot matrix of the seven factors and the percent of runs that lead to an outbreak. To examine the relationships further we calculate the Pearson correlations between each factor. When analyzing the correlations we use the following guidelines for interpreting the coefficients: 0 corresponds to no linear relationship, 0 to 0.3 or 0 to -0.3 corresponds to a weak linear relationship, 0.3 to 0.7 or -0.3 to -0.7 corresponds to a moderate relationship and 0.7 to 1.0 or -0.7 to -1.0 corresponds to a strong linear relationship [36]. The correlations for our model are presented in Table 6. From the scatter plots it can be determined that none of the factors have a clear relationship with the percent of runs that lead to an outbreak. This is further shown in the correlation table, it can be seen that there is no strong correlation between percent of runs that result in an outbreak and any of the factors. This analysis shows that no single characteristic of a town overly impacts the likelihood of an outbreak, but rather that this is governed by the heterogeneity allowed by an ABM and the interactions between agents that are simulated. We can see some relationships between various factors. Population and small areas have a strong positive linear relationship, with a correlation of 0.970. This is as expected as small areas are defined as geographic regions with between 50 to 200 households. A larger population will have more households and thus more small areas. Additionally the percent of unvaccinated agents in the town appears to have a moderate linear relationship with both the percent of students in the town and the population density. The correlation between the percent of unvaccinated agents and students is 0.589 and between the percent of unvaccinated and the density is 0.515. This also makes sense, as the measles vaccination was introduced in 1985 in Ireland. Thus the older population is largely immune due to contracting measles while the younger population would be the ones getting vaccinated. With the same vaccination rates by age across towns, a town with higher percentage of students should have a higher percentage of unvaccinated individuals across the whole town. Table 5 also includes the effective reproductive number, *R*_*e*_, which is the reproductive number, *R*_0_, adjusted to account for immunity in the population. It can be calculated using the following formula, where x is the percent of susceptible individuals in the population:

$$\begin{array}{c} R_{e}=R_{0}*x \end{array}$$ (5)

**Table 6:** Correlation table for percent outbreaks and the other town characteristics.

	Outbreak	Population	Area	Small Areas	Students	Unvaccinated	Transmission	Density
Outbreak	1	0.362	0.312	0.399	0.112	0.208	0.044	0.077
Population	0.362	1	0.203	0.970	0.388	0.462	-0.483	0.718
Area	0.312	0.203	1	0.263	0.001	-0.105	-0.048	-0.347
Small Areas	0.399	0.970	0.263	1	0.251	0.340	-0.462	0.648
Students	0.112	0.388	0.001	0.251	1	0.589	-0.260	0.407
Unvaccinated	0.208	0.462	-0.105	0.340	0.589	1	-0.509	0.515
Transmission	0.044	-0.483	-0.048	-0.462	-0.260	-0.509	1	-0.440
Density	0.077	0.718	-0.347	0.648	0.407	0.515	-0.440	1

#### Towns similar to Schull

We further break our analysis down to towns that have characteristics similar to Schull. Intuitively it would make sense that an infectious disease outbreak in two towns of approximately equal population and area would be similar. However, we hypothesize that this is not always the case and that interactions between both known characteristics that are programmed into the model and other more intangible characteristics that emerge from the model lead to differences in outbreaks. In order to test this, we select twelve of the towns from the previous analysis that have similar population sizes, town area, or both to Schull. Two towns selected are similar in both area and population, four are similar in population and six are similar in area. Table 7 gives the areas, populations, transmission probability and percent of runs that result in an outbreak for the twelve towns plus Schull.

**Table 7:** Percent outbreaks, area and population for each of the 12 selected towns and Schull.

Town	Percent Outbreaks	Population	Area	Transmission
Ardfert	62.5	997	7.97	0.006
Bagenalstown	65.6	3,421	18.00	0.003
Croom	57.5	1,690	18.17	0.004
Kilcock	57.25	6,234	16.40	0.001
Kilkee	66.5	1,037	5.26	0.008
Killadysert	59.3	922	63.96	0.009
Louisburgh	67.5	983	23.30	0.008
Portmagee	57.5	390	16.77	0.023
Rosslare	50.5	2,057	17.90	0.003
Schull	72.5	987	17.03	0.008
Shanagolden	58.3	946	17.79	0.008
Strokestown	74.0	1,003	18.11	0.009
Tramore	73.0	9,548	16.60	0.001

A test of equal proportions is done on the proportions of runs that constitute an outbreak. The null hypothesis of the test is that the proportions in several groups are the same. The test results in a p-value of <0.0001. This results in a rejection of the null hypothesis leading to the conclusion that there are statistical differences between the proportions of outbreaks in the towns. To get a better idea of how area and population affect the outbreaks, towns with area similar to Schull are analysed separately from towns with population similar to Schull and towns with both population and area similar to Schull.

#### Population

The four towns selected with a similar population to Schull but a different area were Ardfert, Kilkee, Killadysert, and Louisburgh. The percent of runs resulting in outbreaks for the four towns range from 59.3% for Killadysert to 67.5% for Louisburgh. All of which are lower than the percent of runs resulting in outbreaks from the Schull model. Running a test of equal proportions for the four towns and Schull gives a p-value of 0.001 resulting in a rejection of the null hypothesis that the proportions are the same. Therefore, it can be concluded that other factors besides population size influence the course of an outbreak.

#### Area

Six towns were selected because they had area similar to Schull. The towns are Croom, Portmagee, Tramore, Bagenalstown, Rosslare and Kilcock. The percent of runs resulting in outbreaks for the six towns range from 50.5% for Rosslare to 73% for Tramore. The percent of runs resulting in outbreaks from the Schull model is in this range, with 72.5% of runs for Schull with more than one agent infected. Running a test of equal proportions for the six towns plus Schull gives a p-value of <0.0001 resulting in the rejection of the null hypothesis, which leads to the conclusion that the area of the town does not determine the size of the outbreak.

#### Area and population

Strokestown and Shanagolden were selected as two towns that shared many characteristics to Schull. Both towns have similar area and population size but additionally have similar age structures. Looking at the percent of students in the population, the group believed to be most susceptible to an outbreak of measles, Schull has 31% students in the town, Strokestown has 31% students and Shanagolden has 33% students. Additionally the proportion of individuals in each town who are not vaccinated and thus susceptible to the infection is compared. Although vaccination rates are constant across towns in the model because the vaccination rates are age specific the overall proportion of vaccinated individuals in a town may vary due to different age structures. Schull, Strokestown and Shanagolden all have 11% of the population who are not vaccinated or not immune.

**Fig 9:**
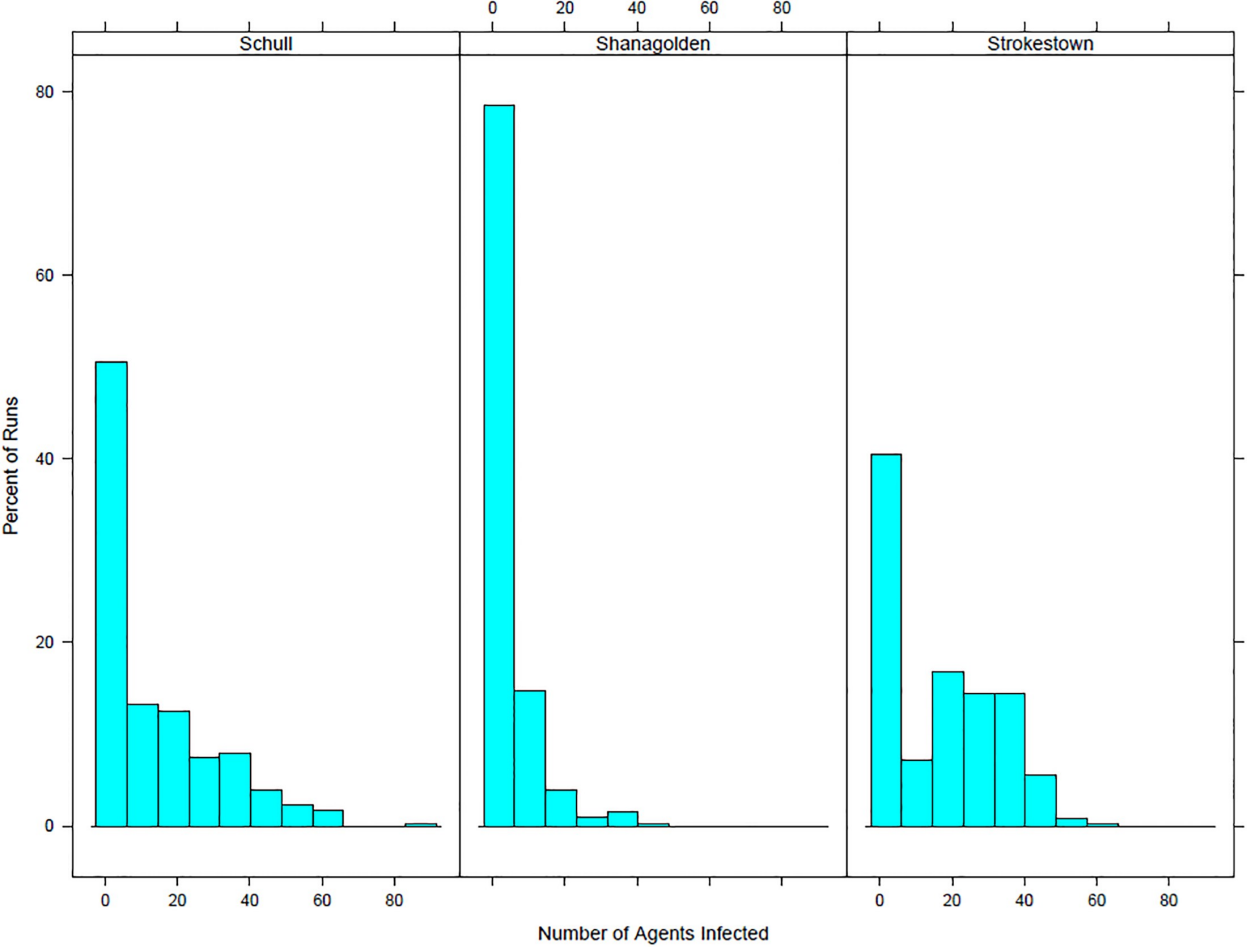
Histograms showing the percent of runs by number of agents infected for Schull, Shanagolden, and Strokestown.

Comparing the results for the three towns, Schull and Strokestown both have similar proportions of outbreak runs, with 72.5% of runs for Schull with more than one agent infected and 74% of runs for Strokestown with more than one agent infected. Shanagolden, however, has different results with 58.3% of runs with more than one agent infected. The differences can be seen in more detail by looking at the overall distrubtion of agents infected by run for the three towns. Fig 9 shows histograms showing the percent of runs by number of agents infected for the three towns.

**Fig 10:**
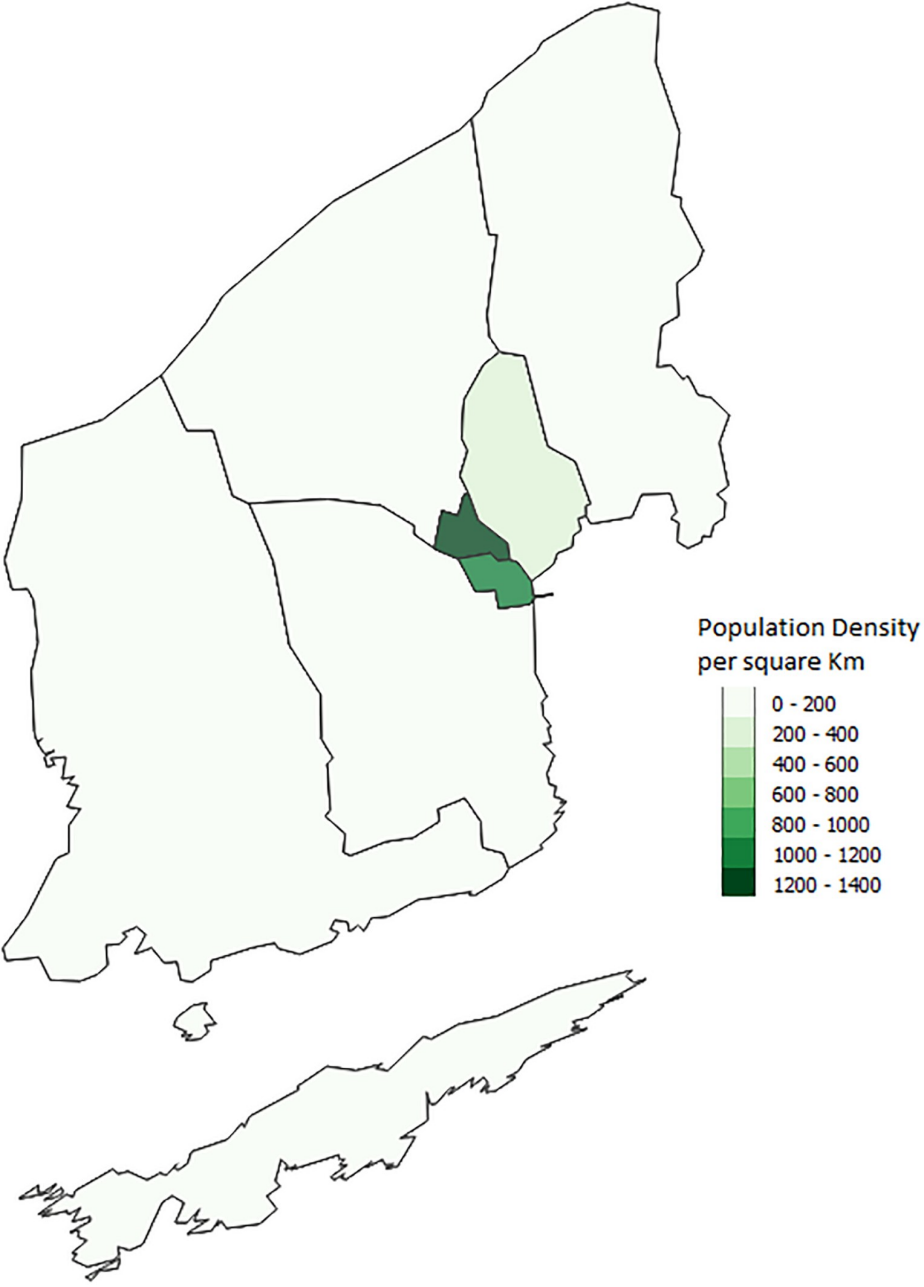
Schull, Ireland. Map showing the population density per sqkm in Schull from the 2011 Census [16].

**Fig 11:**
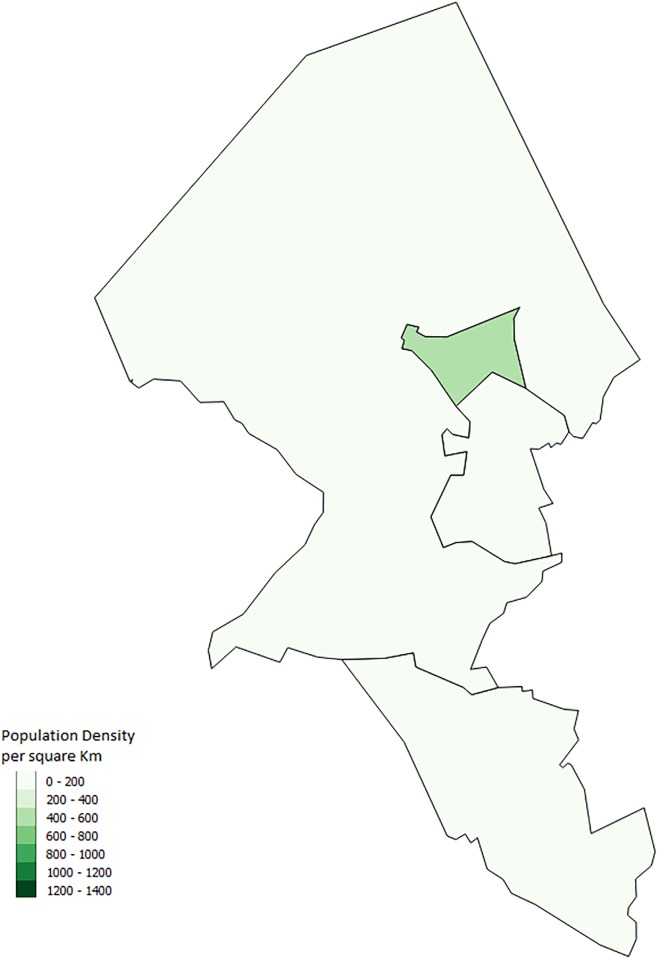
Shanagolden, Ireland. Map showing the population density per sqkm in Shanagolden from the 2011 Census [16].

**Fig 12:**
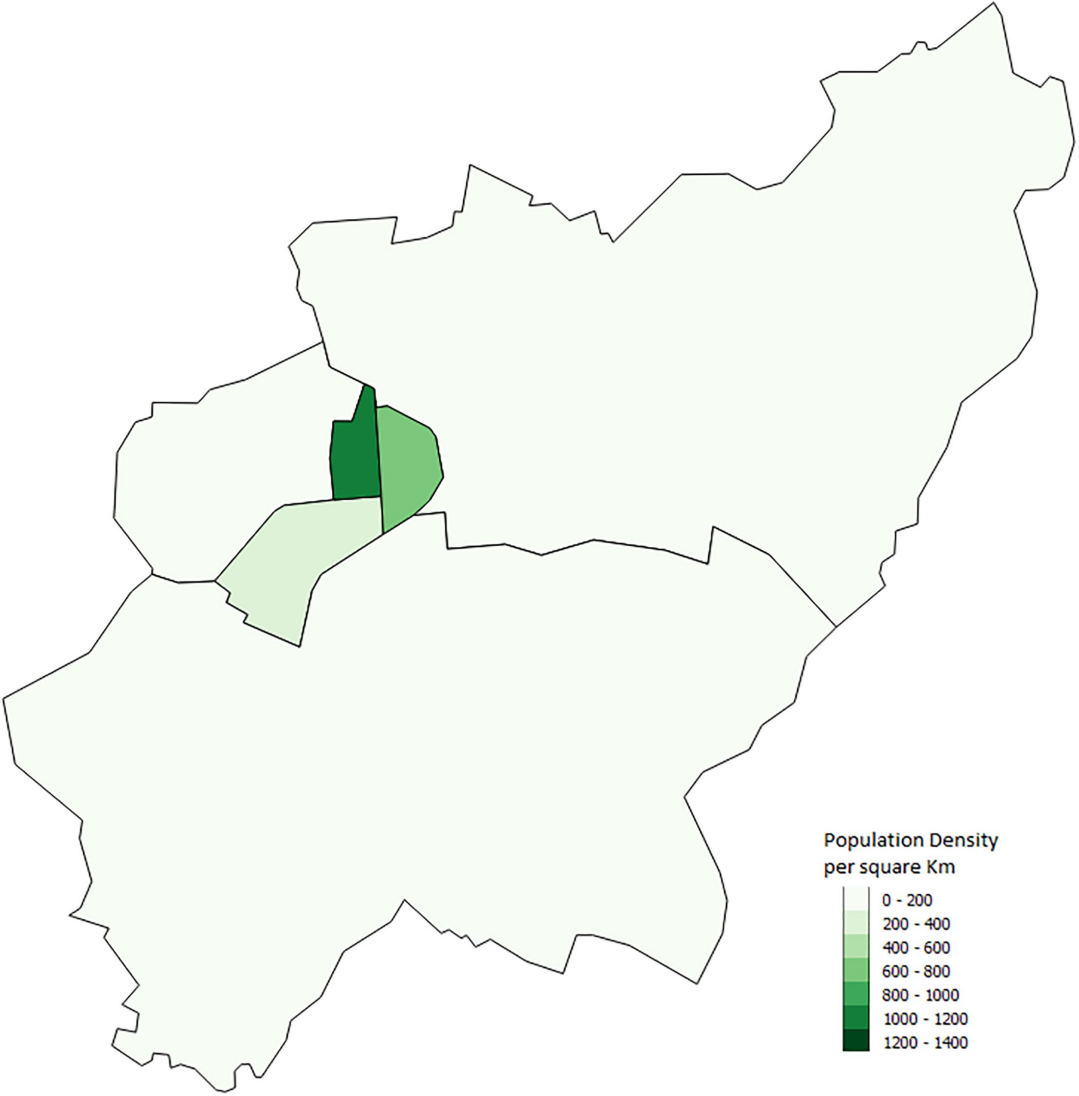
Strokestown, Ireland. Map showing the population density per sqkm in Strokestown from the 2011 Census [16].

Although Schull and Strokestown have similar results, Shanagolden’s lower proportions of outbreaks illustrate that within our model similar towns can have different results. This is to be expected as it is likely that interactions between characteristics and the town layout lead to different outbreaks. These results also emphasize why an agent-based model is important in looking at infectious disease outbreaks. Other modelling methods such as equation based models would not capture these interactions. Although we are not able to determine the exact reason for differences between towns we can make some guesses. One possible difference between the towns is the number of schools. Strokestown has two primary schools while the other two towns have only one. Another possible interaction that could lead to differences has to do with the town layout. Both Strokestown and Schull appear to have a larger town center while Shanagolden appears more spread out. Figs 10, 11 and 12 show the small areas of the towns color coded based on population density. The darker the green color the higher population density. From the figures one can see that both Schull and Strokestown have a few higher density small areas compared to Shanagolden. Both these factors could lead to differences in agent interactions and thus model results.

Based on the results we can conclude that the agent-based model is capturing interactions between the factors and the agents actions and these interactions are what is defining the outbreak. If it was only factors that were programmed into the initial setup of the model that affected the course of the outbreak we would expect a stronger relationship between the percent of runs resulting in an outbreak and the various factors that we discuss.

Further work can be done to improve the model. One area for improvement is scaling. Although looking at small to medium size towns is an interesting case study, a model that encompasses more area and a greater population including movement between towns. Additionally the model can be updated to use the 2016 Census data. The new census data is mostly in the same format as the 2011 Census data so only minimal adjustments to the datasets will be needed. One advantage of the 2016 Census data is workplace zones. They are similar to small areas but are for daytime population so capture where agents work and study. These zones should allow for a more realistic distribution of agents in workplaces than we currently include. Another area of future work could deal with creating a more detailed environment in the model allowing for the model to be run for diseases that have an environmental component such as waterborne illnesses.

## Conclusion

In this paper we have presented an approach to creating an open data-driven ABM for human infectious disease epidemiology. By simply changing a few parameters we are able to quickly and easily adjust the model to simulate an outbreak in any town in Ireland as long as the data for the town is accessible. Similarly if the data is available we are confident that our model can simulate the spread of measles in a non-Irish town. Although we have only presented models for the spread of measles in this paper, with the adjustment of disease modelling parameters, such as probability of infection and exposure time, the model can be adjusted to simulate the spread of any infectious disease that follows the SEIR person to person transmission dynamics.

Using an agent-based model allows one to capture the stochasticity that exists in a real world system. An ABM does not just give one prediction but a range of possible outbreaks that may occur as agents are allowed to make decisions similar to how individuals in the real world will make decisions. Running the ABM multiple times will capture different possible scenarios for the outbreak that are all determined by how the agents interact. For example, when simulating the Schull outbreak, model runs where the outbreak is less severe than the Schull outbreak could be cases where individuals stayed home when infected, and, therefore, did not infect other individuals. Runs where the outbreak is more severe could be due to the lack of inclusion of prevention measures in our model. This is similar to what could happen in a real world scenario, the actions of a few individuals could alter the course of an outbreak, and is important when considering the effects of future outbreaks.

We believe that such as model could eventually be useful as a public health tool. Understanding how different interventions may affect an outbreak or how resilient a population already is can have a major influence on prevention strategies and preparation. Being able to easily model similar outbreaks in different towns can be an important tool in resource allocation, for example deciding where to focus a vaccination campaign when the resources do not exist to focus on every town.

## Appendix 1

### Model description

We use the computer software Netlogo [24] to implement our model. Our model is a data driven ABM for human airborne infectious diseases such as the flu or measles. We have created a simulation with a more general disease model so that it can adjusted for various airborne diseases. The following sections provide a detailed description of the model. We use the ODD format for our model description [25]. It is a standard format used to describe ABMs. The model description is for our current model version.

### Purpose

The purpose of the model is to use openly available data to create an ABM of an infectious disease spread in an Irish town. The model is designed to be transferable to other towns. Specifically, we want to identify factors that might result in towns being more or less susceptible to an outbreak, in particular town density, town population and their interactions along with vaccination rates and socioeconomic makeup of the town.

### Entities, state variables and scales

- **Agents/Individuals:** The model has one type of agent. The agents represent people in the town being modeled. The state variables for each agent including characteristics such as age, gender and economic status. S1 Table shows the full list of variables for each agent.
- **Spatial Units:** Each grid cell or patch in Netlogo represents approximately 111 m^2^ of land. The state variables for each grid cell within the model can be found in S2 Table.
- **Environment:** The model environment is made using data to determine the town boundaries, land use. For town boundaries we use data from the Irish Central Statistics Office (CSO). For accuracy the towns are broken up into small areas. A small area is a technical term used by the CSO to describe the smallest area over which census data is aggregated. A town with small area boundaries can be seen in S1 Fig. The white boundaries are the boundaries of the small areas.
  Environmental variables within the model include time, day of the week and week number. Each time step in the model represents two hours in a day. Seven days will make up one week. The first week in the year is considered week 1. Weeks are tracked to take into account summer vacation for students. Agents determine where they are moving based on the time. The model is affected by the day of the week, as agents will act differently on a weekend versus a weekday. The week number also affects the model; students will not attend school in the summer and will treat everyday as a weekend.

### Process overview and scheduling

The model proceeds in discrete time steps that represent two hour. The model runs until there are no longer any agents who are exposed or immune. Each time step the following submodels are run: clock, move, infect, recover, update-global-variables, update-numcontacts, reset-contacts. Submodels are described in the “Submodels” section.

### Design concepts

- **Basic Principles:** We base the infection part of the model on an SEIR (susceptible, exposed, infected and recovered). It is a model that is widely used within infectious disease modelling. The idea is that when a susceptible agent come into contact with an infected agent there is a certain probability that the agent will become exposed to the disease. This probability is determined using values for *R*_0_ for the disease, basic reproductive number and is defined as the expected number of individuals infected by one infectious individual in a completely susceptible population. It can be broken down into three components, number of contacts per unit time (*c*), the transmission probability per contact (*p*) and the duration of the infectiousness (*d*). The relationship can be seen in Eq 1 [30].
  $$\begin{array}{c} p=\frac{R_{0}}{cd} \end{array}$$ (6)
  Of the four variables in the equation, *R*_0_, *c*, *p*, and *d*, three are known or can be estimated from our model, allowing us to determine the value for *p*. The agent will then progress from exposed to infected and finally to recovered. Agents will come into contact with other agents based on how they move through the environment. The model takes a simplistic approach to agent movement, with agents moving in a straight line between home and workplaces or schools. Agents who do not have a workplace will move randomly in the town to locations in the town center, residential areas or recreational areas. During weekends and summer holidays for students all agents move randomly within the town.
- **Emergence:** The emerging result from the model is the course that the infection takes. Based on the type of agent that is initially infectious, the other agents that come into contact with the infectious agent and how long the contact lasts, patterns can emerge for how an outbreak will spread. For example, if a student is infected compared with an unemployed agent, the student will likely come into contact with more susceptible agents every day it attends school leading to a larger outbreak. Agents decisions to stay home when sick can also have a major effect on how the outbreak occurs.
- **Adaptation:** The current version of the model has little adaptation involved. Agents reproduce observed behaviors based on a set of rules given to them. For example, on weekends agents will move from one location to another a certain percentage of the time. If an agent becomes sick they will adapt their behavior in that they can decide to go about their day as normal or to stay home.
- **Sensing:** As they move through the environment infected agents will sense if other agents that are close to them are susceptible.
- **Interaction:** The model assumes direct interaction between agents. If two agents occupy the same space (both agents are on the same Netlogo patch) it is assumed that they have had some sort of interaction which may lead to the infection of an agent if one is susceptible and one is infected.
- **Stochasticity:** Agent movements in the model are partially random. Weekend movement for all agents, summer movement for students and everyday movements for unemployed, stay at home, retired, and sick and disabled agents are all determined stochastically. Agents will stay where they are x% of the time and (1- x)% of the time they will move to a randomly selected location with a given land use (town center, recreational or residential).
  Additionally stochasticity in the model is seen in the spread of the infectious disease through the population. When an infectious agent comes into contact with a susceptible agent there is a certain probability that determines if the susceptible agent will become exposed. Once exposed the length of time that agent will remain exposed before becoming infectious is determined by a probability distribution. Similarly the length of time an agent stays infectious is determined by a probability distribution.
- **Observation:** Every run of the model data is collected on the number of agents who are susceptible, exposed, infected and recovered at each time step. The output is collected at every time step in order to see how the infection changes over time. Data is also collected on the age of individuals infected along with their work status (student, work, unemployed etc.). The age and work status is only collected the time step that an agent becomes infected. Finally the average number of contacts across all agents in the model is collected.

### Initialization

The town was setup using the small area datasets from the Irish Central Statistics Office (CSO). Small areas are the smallest level of geography that the census data is counted for and are made up of between 50 and 200 homes. Small areas for each town are loaded into the model to determine the boundaries of the town. Zoning data is used to assign patches to a use of open space, town center, community, residential, commercial or mixed. The number of occupied households in each small area can be determined using household tables from the CSO. This number is then used to randomly generate the correct number of household within each small area and households are placed in residential patches. Patches with a zone of commercial, town center or mixed can be a workplace. School location datasets are used to find the locations of the schools in the town. Agents are added to the town based on the closest census data to the year being modelled. Census data is produced every five years with the two most recent datasets for the 2011 and 2016 census. If we were modelling an outbreak from 2012 the 2011 census data would be used. However, if the outbreak was in 2014, the 2016 census data would be used. The number of agents in the simulation will be the number of people living in the town. The following steps are used in populating the town with agents and are performed for each individual small area:

- Each household is assigned a type (single, couple, couple plus others, couple with children, couple with children plus others, single parent, single parent plus others or other).
- Adults are added into each household. One agent is added to households with types single, single parent and other. Two agents are added to the households with type couple.
- Adults in each household are assigned a sex and age based on a probability distribution determined from the CSO census age, sex tables for the relevant small area.
  - The age categories provided by the CSO are by year until 19 after which ages are reported in ranges of five years, for example ages 20-24 or ages 60-64, and then anyone over 85 is combined into one age bracket. To have all ages represented in our model in each age bracket we randomly assign individuals one of the five ages represented in that bracket.
  - Couples are assigned opposite genders and ages within 10 years of each other. The youngest age given to any agent in a couple in the same household is 18.
- If a household type includes children a probability distribution determined from the relevant census data is used to determine if all children in the house are under 15, over 15, or both over and under 15.
- Data from the family units with children by size and age of children table is used to determine the probability that each household with type child has 1, 2, 3, 4 or 5 children in the household.
- Children are added into each household.
- Children are assigned a sex and age based on a probability distribution extracted from the relevant census data and the type of children the household is assigned to (under 15, over 15 or both under and over 15).
- If the total number of agents populating a small area is not equal to the total number of agents who should be in the area based on the CSO data, additional agents are added and randomly assigned to households of types couple plus others, couple with children plus others, single parent plus others or other.
- All agents are assigned an economic status based on CSO data.
  - Agents over 65 are assigned to retired.
  - Agents between the ages of 5 and 14 are assigned to student.
  - Agents between the ages of 15 and 18 are first assigned to student. If there are more agents aged 15 to 18 in the small area than the number of students in the same age categories then the agents are assigned to looking for first job. If there are still more agents aged 15 to 18 they are then assigned an economic status of work, unemployed or sick/disabled following the distribution for these categories for the relevant small area.
- Adult agents under 65 are assigned to work, looking for first job, unemployed, sick/disabled or stay at home following the distribution for these categories for the relevant small area. Agents are only assigned to stay at home if they are part of a couple.
- Agents under the age of 5 are assigned to student if they have no stay at home parent. If they have a stay at home parent then a probability determines if the agent will be assigned to student.
- Agents with an economic status of work are randomly assigned to one of the work-places in town.
- Agents with the economic status of student are assigned to the closest school that matches their age
  - Students younger than 4 are assigned to a preschool.
  - Students with ages 4 to 12 are assigned to a primary school.
  - Students 13 and older are assigned to a secondary school.
- Agents can be assigned a social class.
  - Agents with an economic status of work are given one of the following social classes: Professional Workers, Managerial and Technical, Non-Manual,Skilled Manual, Semi-Skilled, or Unskilled.
  - Agents who are retired are given a social class of retired and agents who are unemployed, sick/disabled, looking for first job, or stay-at-home are given the social class other.
  - Agents who are younger than 18 are not given a social class.
  - Households are assigned a household social class. This is randomly selected from one of the adults in the household. All agents in a household, including children, will have the same household social class.
- If vaccinations are included in the model agents are given an immunity level based on the disease being modeled and vaccination data for that disease. Irish vaccination data is used to determine the percentage of each age group that have received vaccinations for the infectious disease being modelled. For example, if 90% of 1 year olds in Ireland had been given the MMR vaccination in 2011 and we are running a model for 2012, we give each agent in the model with an age of 2 a 90% chance of having been vaccinated. If an agent is vaccinated they are given a 97% chance of being immune to the disease. This takes into account vaccination failure and is based on the vaccine effectiveness rate for measles [26]. Half of the agents with age less than 1 are given immunity to a disease to mimic passive immunity infants receive from their mothers [27]. For any agents that have an age corresponding to a vaccination year not in our data we give a 99% chance of being immune. Prior to vaccination campaigns the majority of the population would have either had or been exposed to childhood diseases, such as measles, leaving them immune in later life. If socioeconomic status is used in the model, the percent of individuals receiving vaccinations is adjusted based on the household socioeconomic status of the individual.
- Finally, a given number of agents are given the status of infected.

### Input data

The model does not use input data to represent time-varying processes.

### Submodels

- **Clock:** The clock submodel keeps track of the time, day and week of the model. The time is the determined as the modulus of ticks and 12. When the time goes back to 0 the day is increased by one. If the day is 8, the week is increased by one and the day goes back to 1.
- **Move:** In the model agents use straight-line transportation. Agents will move between their home and destination in a straight line following the most direct route. Although this is a naive model, for small towns where distances travelled are short, such as those discussed in this paper, it is effective.
  - Agents who are working leave their home on the fourth time step of the day, which would be equivalent to between 8am and 9am, arrive at work over one time step, spend 4 time steps (8 hours) at work and then return home.
  - Students also leave on the fourth time step but only spend 3 time steps (6 hours) at school.
  - Stay at home agents who have children in primary school travel with their children to school on the 4th time step and then return home during the same time step. Between the fourth and seventh time step (when students return home from school) stay at home agents move randomly throughout the town: at each step if an agent is at home they have a 50% chance of staying at home. If not at home, an agent has a 50% chance of picking a new destination in town and moving there. At the 7th time step of the day the stay at home agents will go to their child’s school and then travel home.
  - Stay at home agents who do not have a child in primary school move randomly throughout the town between the fourth time step and the seventh time step the same way stay at home parents move when they are not travelling with their children to school or home.
  - Agents younger than 4 who are not assigned to a preschool move with their stay at home parent throughout the day.
  - Agents who are unemployed, looking for their first job, retired or sick/disabled move randomly throughout the town between the 4th and 10th time steps of the day.
  - If an agent is infected with the disease simulated within a model then their behaviour is affected. Infected agents have a certain probability of staying home. If they are working, the agents will stay home 30% of the time. Students will stay home 70% of the time. Unemployed agents will stay home 75% of the time, and stay at home agents with primary school children will stay at home 10% of the time when accompanying children to school and 50% of the time when moving around town. Stay at home agents with non-primary schoolchildren will stay at home 50% of the time.
  - All agents will move randomly through the town on the weekends.
- **Infect:** When an infected agent comes into contact with a susceptible agent, the infected agent will determine if they will infect the susceptible agent based on the probability of infection, a variable chosen at the start of the simulation. If the infected agent determines it will infect the susceptible agent, the susceptible agent will change their health status from susceptible to exposed.
- **Recover:** An exposed agent will use a probability distribution to determine the number of time steps it will stay exposed before it becomes infected. Similarly, when the agent switches from exposed to infected the agent will use a probability distribution to determine the number of time steps before they are recovered/immune. Once an agent has recovered they cannot become infected again.
- **Update global variables:** At the end of each time step, all global variables are updated. The counts and percent of susceptible, exposed, infected and recovered agents are all calculated. The average number of contacts across all agents in the model is calculated by taking the average of each agent’s contacts.
- **Update numcontacts:** As agents move through the environment they keep track of every agent, they come into contact with (agents on the same patch of the environment). At the end of the time step, the agents will calculate the number of unique contacts they have had and calculate an average number of contacts they have had during the simulation. An overall average for number of contacts across all agents is then taken.
- **Reset contacts:** After the average number of contacts has been calculated, the contacts each agent has had is reset to 0 so that they can calculate a new average for the next tick.

## Supporting information

S1 Table State variables for agents in the model. (XLSX)

S2 Table State variables for grid cells in the model. (XLSX)

S1 Fig Image of the model environment for Schull, Ireland. The white boarders are the boarders of the small areas that make up the town.
(TIF)
