# Correction: An open-data-driven agent-based model to simulate infectious disease outbreaks

**DOI:** 10.1371/journal.pone.0211245

**Published:** 2019-01-17

**Authors:** Elizabeth Hunter, Brian Mac Namee, John Kelleher

In the Author Contributions section, John Kelleher should be listed as one of the persons who conceptualized the study.
