# Supplementary figures and images for: An open-data-driven agent-based model to simulate infectious disease outbreaks
Source: PLoS One. 2018 Dec 19;13(12):e0208775. doi: 10.1371/journal.pone.0208775 (PMC6300276; doi:10.1371/journal.pone.0208775)

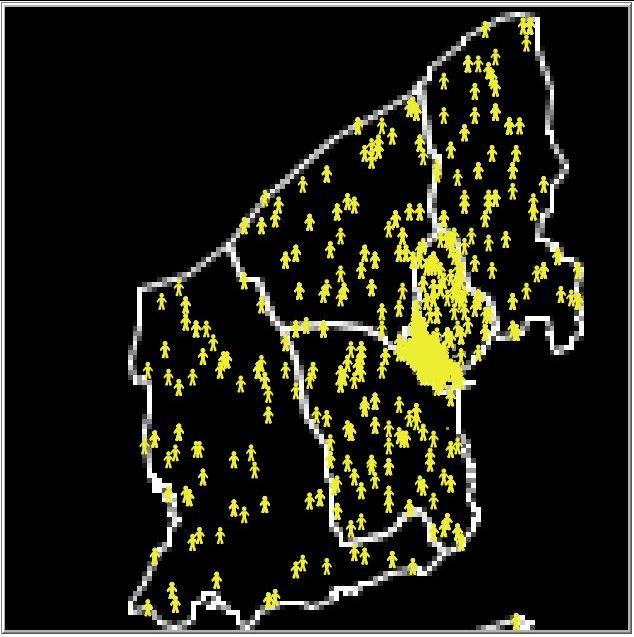

Supplement: S1 Fig — The white boarders are the boarders of the small areas that make up the town. (TIF) [file pone.0208775.s003.tif]
